# A numerical and experimental approach to oil recovery performances during combined xanthan gum and carbon dioxide flooding

**DOI:** 10.1038/s41598-026-49640-7

**Published:** 2026-05-01

**Authors:** A. N. El-hoshoudy, E. M. Mansour

**Affiliations:** 1 PVT Lab, Production Department, Egyptian Petroleum Research Institute, Cairo, 11727 Egypt; 2 PVT Service Center, Egyptian Petroleum Research Institute, Cairo, 11727 Egypt

**Keywords:** EOR, Xanthan gum, CO_2_ gas flooding, Mobility control, Synergistic injection, CMG modelling, Energy science and technology, Engineering

## Abstract

Carbon dioxide enhanced oil recovery (CO_2_-EOR) offers dual benefits of increasing hydrocarbon production and sequestering anthropogenic CO_2_; however, conventional CO_2_ flooding suffers from unfavorable mobility ratios and premature gas breakthrough, while polymer flooding alone faces challenges in high-salinity and high-temperature reservoirs. This study experimentally and numerically investigates the synergistic impact of combining xanthan gum polymer with CO_2_ injection to maximize oil recovery. We conducted laboratory coreflood experiments on packed sandstone (porosity 22%, permeability 1000 mD) saturated with 31°API crude oil (6.0 cP at 70 °C), testing polymer concentrations of 1.0, 1.5, 2.0, and 2.5 g/L. We evaluated five flooding scenarios: water flooding (baseline), CO_2_ flooding alone, polymer flooding alone, polymer (1.5 g/L) followed by CO_2_, and CO_2_ followed by polymer (1.5 g/L). We then upscaled experimental findings using CMG-IMEX reservoir simulator to field scale. Water flooding recovered 70.0% of original oil in place (OOIP). CO_2_ flooding alone increased recovery to 83.3% OOIP. Polymer flooding alone achieved maximum recovery of 81.3% OOIP at optimal concentration (1.5 g/L), while lower (1.0 g/L) and higher (2.5 g/L) concentrations yielded 79% and 73.5% OOIP, respectively. Sequential injection significantly outperformed single methods: polymer (1.5 g/L) followed by CO_2_ achieved 89.3% OOIP, while CO_2_ followed by polymer (1.5 g/L) achieved the highest recovery of 94.3% OOIP. An incremental gain of 24.3% over water flooding and 11.0% over CO_2_ flooding alone. The combined polymer-CO_2_ flooding system delivers superior oil recovery compared with individual processes. The optimal strategy-CO_2_ injection followed by 1.5 g/L xanthan gum polymer-maximizes both microscopic displacement efficiency (CO_2_-driven oil swelling and viscosity reduction) and macroscopic sweep efficiency (polymer-driven mobility control). This hybrid approach represents a technically viable and economically promising EOR strategy for mature sandstone reservoirs while contributing to sustainable carbon management.

**Supplementary Information:**

The online version contains supplementary material available at 10.1038/s41598-026-49640-7.

## Introduction

The rapid depletion of conventional oil reserves, coupled with the growing global demand for energy, has intensified the importance of enhanced oil recovery (EOR) technologies for sustaining hydrocarbon production from mature reservoirs^1^. Chemical flooding is a widely used method in the petroleum industry, particularly in enhanced oil recovery (EOR), to maximize crude oil extraction from reservoirs^2^. Since oil resources are becoming increasingly scarce and oil prices continue to rise, major petroleum companies have shifted their focus toward advanced recovery methods that can maximize the recovery factor of existing fields and extend their productive lifespan^3^. The recovery of hydrocarbons during a reservoir’s life cycle can generally be divided into three stages: primary, secondary, and tertiary (enhanced) recovery^4,5^. During the primary recovery phase, oil is produced by utilizing the reservoir’s natural energy, including rock and fluid expansion, gas-cap drive, water influx, and gravity drainage^6^. Secondary recovery typically involves water or gas injection to maintain reservoir pressure and displace additional oil that cannot be produced naturally^7^. Once the energy of these two stages becomes insufficient, tertiary or enhanced oil recovery (EOR) methods are employed to recover the remaining oil through the injection of specialized fluids such as steam, miscible gases, surfactants, polymers, or microbial solutions^8,9^. Among the various EOR techniques, miscible/immiscible carbon dioxide (CO_2_) injection has been widely recognized for its dual benefits of improving oil recovery and facilitating carbon sequestration. CO_2_-EOR has been practiced for more than four decades and currently accounts for approximately 43% of total EOR production in the United States. CO_2_ injection in this study was conducted under immiscible conditions (injection pressure 400 psi, below the estimated MMP of ~ 1800–2000 psi for this crude oil). Nevertheless, partial CO_2_ dissolution into the oil phase occurred, contributing to oil swelling and viscosity reduction. The mechanism of CO_2_ flooding involves contacting and mobilizing residual oil through improved microscopic displacement and sweep efficiencies^10^. Depending on the reservoir pressure, temperature, and oil composition, CO_2_ injection can occur in either miscible or immiscible modes, with the miscible mode typically yielding higher recovery due to the dissolution of CO_2_ in crude oil, which reduces oil viscosity and interfacial tension^11^. CO_2_ injection in enhanced oil recovery is strongly governed by phase behavior and reservoir conditions. A critical parameter is the minimum miscibility pressure (MMP), which depends on crude oil composition (particularly the C^5+^ fraction), reservoir temperature, and CO_2_ purity, with lighter oils and higher-purity CO_2_ generally resulting in lower MMP values. When reservoir pressure is below the MMP, immiscible CO_2_ flooding prevails, where recovery is driven by mechanisms such as oil swelling, viscosity reduction, and solution gas drive rather than full miscibility. However, the relatively low density and viscosity of CO_2_ can lead to gravity override and viscous fingering, reducing sweep efficiency and causing early breakthrough. These challenges highlight the need for effective mobility control strategies to enhance the performance of CO_2_-based EOR processes.

Polymer flooding, on the other hand, has been extensively applied in viscous oil reservoirs worldwide as a cost-effective and reliable method to improve the macroscopic sweep efficiency^12^. The principle of polymer flooding lies in reducing the mobility ratio between the injected water and oil by increasing the viscosity of the injected water. This enhances both vertical and areal sweep efficiencies, thereby improving the overall recovery factor^13^. Xanthan gum, a biopolymer produced by *Xanthomonas campestris*, has been widely studied for this purpose due to its high viscosity at low concentrations, shear-thinning behavior, salinity tolerance, and environmental compatibility^12,14^. Polymer flooding efficiency is governed by several interrelated factors. Elevated temperatures can accelerate thermal and oxidative degradation, leading to a reduction in polymer viscosity due to increased molecular mobility^15^. High salinity, particularly the presence of divalent cations such as Ca^2^⁺ and Mg^2^⁺ , reduces polymer effectiveness by screening electrostatic repulsion and inducing chain coiling, which lowers solution viscosity. Polymer adsorption and mechanical retention within porous media decrease the effective polymer concentration and may impair permeability^16^. In contrast, shear-thinning behavior enhances injectivity at higher flow rates near the wellbore while maintaining sufficient viscosity within the reservoir to ensure effective mobility control. Considerable research has been devoted to improving the efficiency of combined polymer and carbon dioxide (CO_2_) flooding for enhanced oil recovery (EOR). Early efforts were largely focused on identifying polymers or copolymers capable of increasing the viscosity of CO_2_ to improve its mobility control. Bullen et al. (1987)^17^ patented a liquid CO_2_ formulation containing a polycarbonate copolymer (1.5–3 wt.%, Mw 20,000–150,000 g/mol), reporting a significant increase in CO_2_ viscosity under pressures of 1450–3625 psia and at 22 °C. Dandge et al. (1987) ^18^ evaluated the solubility of 53 commercial polymers in CO_2_ and found only 18 to be partially soluble (0.24–1.1 wt.% at 20–58 °C and 1700–3100 psia). Their results, based on windowed falling-cylinder experiments, showed less than a 25% increase in viscosity, largely attributed to the polymers’ low molecular weights (Mw < 1000 g/mol). Subsequent work by Bae et al. (1993, 1995) ^19,20^ explored the use of silicone polymers, such as polydimethylsiloxane (PDMS), in combination with organic co-solvents. They observed that the addition of 4 wt.% PDMS (Mw 197,000 g/mol) with 20 wt.% toluene to CO_2_ (76 wt.%) resulted in a 30-fold increase in viscosity at 4500 psia and 50 °C. This study demonstrated the potential of co-solvent-assisted polymer thickening of CO_2_. Likewise, Huang et al. (2000) ^21^ produced associative copolymers derived from PFA to lower the polymer concentration needed for effective thickening. Efforts to develop more cost-effective alternatives led Tapriyal et al. (2008) ^22^ to introduce PolyBOVA, a non-fluorinated benzoyl-vinyl acetate polymer (3.7 wt.%, Mw 1,400,000 g/mol), which achieved an 80% increase in CO_2_ viscosity at 9300 psia and 25 °C. PolyBOVA provided a promising non-fluorinated option for CO_2_ thickening, though it required higher pressures for dissolution. Zhang et al. (2011) ^23^ revisited earlier formulations from Heller’s research and reported a dramatic viscosity increase (1300–1400%) using low-molecular-weight polymers such as poly(vinyl ethyl ether) (PVEE) and poly(1-decene) (P1D). However, these findings were later disputed by Lee et al. (2014) ^24^, through using a falling-ball viscometer to confirm that these polymers failed to replicate the reported thickening behavior, emphasizing that low-molecular-weight polymers were ineffective CO_2_ thickeners. Parallel to these laboratory studies, Rousseau et al. (2012)^25^ examined the role of polymer-oil-rock interactions in controlling CO_2_ mobility through coreflood experiments and modeling. Their findings underscored that polymer-assisted CO_2_ systems could enhance sweep efficiency but that polymer type, solubility, and rock wettability significantly influenced the outcome. Lee et al., 2014^24^ evaluated CO_2_ foam flooding following polymer flooding in homogeneous sandpack cores under reservoir conditions. Their experiments demonstrated that CO_2_ foam flooding achieved substantial incremental oil recovery even after polymer flooding, with high-quality foam (indicated by greater pressure gradients) improving sweep efficiency and residual oil displacement^26^. Building on this, Zaberi, 2017^27^ synthesized a fluorinated polymer, poly(1,1-dihydroperfluorooctyl acrylate) (PFA), which dissolved directly in CO_2_ at 3.7 wt.% without co-solvents and enhanced CO_2_ viscosity by a factor of 2.5 under similar pressure and temperature conditions. The development of PFA represented a major advancement, highlighting that high-molecular-weight fluoropolymers could effectively dissolve in CO_2_. Yang et al. (2018)^28^ proposed a polymer-alternating-gas (PAG) process combining polymer flooding with CO_2_ injection. Using a reservoir model from the Liaohe Oilfield, they reported an additional oil recovery exceeding 10% compared with conventional EOR techniques, along with favorable economic feasibility. This study marked one of the first applications of PAG in highly heterogeneous heavy oil reservoirs. More recently, Chen et al. (2023)^29^ investigated polymer-assisted CO_2_ flooding for developing low-permeability reservoirs and achieving net-zero CO_2_ emissions. They optimized a temperature-resistant polymer surfactant (TRPS) with strong rheological and salt-resistant properties and identified an optimal concentration of 1000 mg/L. Coreflood tests showed that TRPS-assisted CO_2_ flooding improved oil recovery by 8.21% compared with water-assisted CO_2_ flooding, confirming its superior injectivity and profile control. Collectively, these studies reveal that the viscosity of CO_2_ can indeed be enhanced using high-molecular-weight polymers or polymer–surfactant systems. However, many laboratory successes rely on the presence of organic co-solvents (typically 10% of the total mixture), which render field-scale implementation economically and operationally challenging. The continued evolution of CO_2_-thickening research from early synthetic polymers to modern water-soluble and environmentally benign biopolymers reflects the ongoing pursuit of technically feasible and sustainable hybrid polymer-CO_2_ EOR solutions^27,30,31^. It is important to emphasize that the decision to implement EOR methods, including the hybrid polymer-CO_2_ approach investigated here, must be preceded by thorough technical and economic screening. Reservoir screening criteria for EOR methods typically consider oil properties (density, viscosity, composition), reservoir characteristics (depth, temperature, permeability, heterogeneity), and remaining oil saturation^32^. Before pursuing tertiary methods, operators should optimize secondary recovery strategies, including infill drilling, pattern realignment, and water-alternating-gas (WAG) injection, which may recover additional oil at lower cost^33^. The presence of residual oil after waterflooding does not automatically justify EOR implementation; the incremental oil recovery must be weighed against the additional capital and operating costs, including polymer procurement, CO_2_ capture and compression, and facility modifications. Economic viability depends on oil price, chemical costs, CO_2_ availability, and regulatory incentives for carbon sequestration^34^. The experimental results presented here establish technical feasibility; field application would require detailed reservoir simulation and economic analysis specific to the target reservoir.

Recent research has explored the integration of CO_2_ injection with polymer flooding to exploit the complementary strengths of both methods. In such hybrid processes, polymer solutions are injected alongside or alternately with CO_2_, effectively replacing the conventional water-alternating-gas (WAG) approach^35^. The presence of polymer improves the mobility ratio, stabilizes CO_2_ flow, and delays gas breakthrough, while CO_2_ enhances oil swelling and reduces the residual oil saturation^36^. Moreover, supercritical CO_2_ can offset the shortcomings of polymer flooding by promoting more uniform oil saturation and reducing the overall polymer consumption^37^. This combined process not only enhances recovery efficiency but also contributes to reduced greenhouse gas emissions and improved CO_2_ utilization efficiency^38^. Further developments have focused on using environmentally friendly and biodegradable polymers that can degrade naturally under reservoir conditions, minimizing ecological impact^39^. These polymers are typically water-soluble and compatible with CO_2_ without requiring high temperature, pressure, or catalytic activation. Early efforts to modify the viscosity of CO_2_ concentrated on identifying suitable thickening agents or polymers soluble in hydrocarbons, due to CO_2_’s limited miscibility with water^40^. While nonpolar organic polymers were initially tested, water-soluble polymers like xanthan gum have shown superior compatibility and injectivity in practical EOR applications^41,42^. Despite the growing body of research, the mechanistic understanding of xanthan-CO_2_ co-injection and its performance under realistic reservoir conditions remains limited^43^. Therefore, this study aims to investigate the synergistic effects of combining xanthan gum polymer and carbon dioxide flooding for enhanced oil recovery. Laboratory coreflood experiments were conducted to evaluate the influence of polymer concentration, injection strategy, and CO_2_ phase behavior on oil displacement efficiency, mobility control, and sweep performance^44^. The findings provide new insights into optimizing hybrid polymer-CO_2_ injection schemes for efficient and sustainable EOR operations. Consequently, The novelty of this work lies in: (1) systematic optimization of xanthan gum concentration (1.0–2.5 g/L) for hybrid CO_2_-polymer flooding, identifying 1.5 g/L as optimal; (2) comparative evaluation of injection sequence (polymer → CO_2_ vs. CO_2_ → polymer), demonstrating that CO_2_ followed by polymer yields highest recovery (94.3% OOIP); (3) integration of detailed viscoelastic characterization (G′/G″ analysis) with coreflood performance to establish rheological criteria for effective CO_2_ mobility control; and (4) validation of experimental trends through field-scale CMG-IMEX simulations, bridging laboratory and reservoir scales. This combined experimental–numerical workflow provides new insight into the optimal integration of xanthan polymer flooding with CO_2_ injection for improved mobility control and sweep efficiency.

### Mechanisms of polymer–CO_2_ interaction

The efficiency of polymer-CO_2_ combined flooding in enhanced oil recovery depends largely on the physicochemical interactions between the polymer, CO_2_, reservoir fluids, and the rock matrix^45^. Understanding these mechanisms is essential for optimizing mobility control, enhancing sweep efficiency, and improving the overall displacement of residual oil^46^. The interactions can be broadly classified into three categories: **(1) thermodynamic interactions between CO₂ and polymer molecules, (2) rheological and interfacial effects**, and **(3) reservoir-scale mobility and sweep dynamics**^47^. At the molecular level, the solubility and compatibility of polymers in CO_2_ determine their ability to alter the viscosity of the injected phase. Supercritical CO_2_ is a non-polar, quadrupolar solvent with relatively low polarizability and poor hydrogen-bonding capacity^48^. As a result, it interacts more favorably with non-polar or fluorinated polymers than with polar, water-soluble polymers. The introduction of functional groups such as fluorinated or siloxane moieties into the polymer backbone enhances CO_2_–polymer affinity through weak dipole and dispersion forces, improving solubility and allowing for moderate viscosity enhancement^10^. Conversely, water-soluble biopolymers such as xanthan gum do not dissolve in CO_2_ but can influence its mobility indirectly by modifying the aqueous phase rheology, reducing water–gas mobility contrast, and stabilizing dispersed CO_2_ phases^49,50^. Rheologically, polymer addition increases the viscosity of the aqueous phase and modifies the flow behavior from Newtonian to pseudoplastic or shear-thinning. This change in rheology reduces the mobility ratio between the displacing and displaced fluids, leading to improved macroscopic sweep efficiency^51^. In CO_2_-polymer systems, this effect manifests in two principal ways. First, when polymer and CO_2_ are injected alternately or simultaneously, the polymer solution creates a viscous barrier that restricts CO_2_ channeling through high-permeability zones, thus promoting a more uniform flood front^47^. Second, under certain conditions, the interaction of CO_2_ with polymer-stabilized surfactant films can generate stable foams or emulsions that further enhance gas confinement and reduce mobility^9,52^. Such CO_2_-polymer foams exhibit improved stability due to the high viscosity of the aqueous phase and the elastic nature of the polymer films surrounding gas bubbles^53^. It should be noted that while CO_2_-polymer foams can be stabilized by surfactant films in systems where surfactants are present (either naturally in the crude oil or co-injected), the experiments in this study did not include added surfactants. However, xanthan gum itself possesses some surface activity and can stabilize CO_2_-in-water emulsions through its viscoelastic interfacial film formation^54^.

From a reservoir-scale perspective, the synergy between polymer and CO_2_ injection arises from complementary displacement mechanisms. CO_2_ injection reduces oil viscosity and interfacial tension through dissolution and oil swelling, thereby improving microscopic displacement efficiency. Meanwhile, polymer injection increases macroscopic sweep efficiency by improving mobility control and reducing viscous fingering. The combined process therefore achieves higher total recovery than either method alone, as CO_2_ compensates for the limitations of polymer flooding (such as poor displacement in low-permeability zones) and the polymer mitigates early CO_2_ breakthrough in highly permeable channels^55,56^. Additionally, the polymer–CO_2_ alternating gas (PAG) and polymer-assisted CO_2_ flooding (PACF) techniques have demonstrated improved displacement uniformity and pressure profiles in both simulation and experimental studies^57^. The PAG process involves alternating slugs of polymer solution and CO_2_ to maintain favorable mobility ratios throughout the reservoir, while PACF entails co-injection or surfactant-enhanced polymer formulations that stabilize CO_2_ foams^58^. The efficiency of these processes depends on parameters such as polymer concentration, CO_2_ injection pressure, temperature, salinity, and rock wettability. The mechanisms of polymer-CO_2_ interaction operate through a combination of molecular, rheological, and flow-scale effects that collectively improve displacement and sweep efficiencies^59^. By modifying CO_2_ mobility and promoting more uniform flooding patterns, the hybrid process can yield substantial incremental oil recovery^60–62^.

## Materials and characterization

Commercial-grade xanthan gum polymer supplied by a local vendor was used without further purification. Analytical-grade calcium chloride (CaCl_2_), magnesium chloride (MgCl_2_), and sodium chloride (NaCl) were employed to prepare a synthetic brine representative of sandstone formation water, with a total salinity of 135,000 ppm. Xanthan solutions were prepared in deionized water by gradually adding xanthan gum (3 g/L) to the brine per API-RP-63, gently stirred at 500 rpm to prevent degradation, and stored airtight to avoid evaporation or contamination. The crude oil used was dead oil obtained from production tanks, having an API gravity of 31° and a viscosity of 6.0 cP, density of 0.835 g/cc at a reservoir temperature of 70 ˚C, and was degassed and filtered before use. The crude oil (31°API, 6.0 cP at 70°C) is classified as light-to-medium oil according to standard API gravity ranges, making it suitable for CO_2_ flooding applications where oil viscosity is sufficiently low to allow favorable mobility conditions. The composition analysis of the stock tank oil is provided in supplementary material (Table S1). High-purity CO_2_ gas (99.98% CO_2_ and 0.02% N_2_) was used to simulate injection gas conditions in EOR processes^63,64^. All solutions were prepared using deionized water, and polymer solutions were thoroughly mixed at ambient temperature until complete dissolution. The prepared fluids were stored in sealed containers to prevent evaporation, contamination, and CO_2_ loss before testing. Xanthan gum characterization was conducted through spectroscopic analysis, such as (FTIR), (AFM), (TGA), and (NMR) analysis, as detailed in our previous reports^12,65,66^. The xanthan gum used in this study has an average molecular weight (Mw) of approximately 2.5 × 10⁶ g/mol, as determined by gel permeation chromatography (GPC), and an intrinsic viscosity [η] of 4500 mL/g in 0.1 M NaCl solution at 25 °C, consistent with values reported for high-molecular-weight xanthan biopolymers^67,68^. The rheological behavior of the polymer solutions was evaluated using an Anton Paar RheoCompass™ MCR 102e rheometer under ambient and reservoir conditions. Steady shear and frequency sweep tests were conducted to determine viscosity–shear relationships and to characterize the viscoelastic properties through the storage (G′) and loss (G″) moduli^14^.

### Sand pack flooding

Flooding experiments were carried out using a homogeneous linear sand-pack model at 70 °C to evaluate the performance of xanthan gum polymer at concentrations of 1.0, 1.5, 2.0, and 2.5 g/L, in combination with CO_2_ injection. The sand pack was thoroughly cleaned using a Soxhlet extractor to remove residual salts and impurities, followed by oven drying at 60 °C under controlled low-humidity conditions. Key petrophysical properties, including porosity and permeability, were determined from bulk and pore volume measurements. The experimental setup, consistent with that described in literature^69^, enabled controlled injection of synthetic brine, crude oil, polymer solutions, and CO_2_. Initial brine saturation and absolute permeability measurements were conducted at 20 °C under an injection pressure of 400 psi and an overburden pressure of 4000 psi. Crude oil was subsequently injected until connate water saturation (S_wi_) and effective oil permeability at S_wi_ (K_o_@S_wi_) were established. These parameters were then used to calculate the original oil in place (OOIP) based on Equations (1–4).

1 $$V_{B} = \pi r^{2} L$$

2 $$\phi = \frac{{V_{p} }}{{V_{B} }}$$

3 $$S_{wi} = \frac{{\left( {V_{B} - V_{w} } \right)}}{{V_{B} }}$$

4 $$OOIP = V_{p} \times \left( {1 - S_{wi} } \right)$$

The sand pack model had a length of 62.8 cm and an internal diameter of 2.5 cm, corresponding to a cross-sectional area of 19.6 cm^2^ and a bulk volume of 1368 cm^3 69^. The measured pore volume was approximately 300.96 cm^3^, yielding an average porosity of about 22% and a permeability of approximately 1000 mD. Absolute permeability was obtained through brine injection at multiple flow rates, while porosity was determined gravimetrically by comparing dry and fully saturated weights. The model consisted of a stainless-steel core holder packed with 100–200 mesh sand. A confining pressure of 4000 psi was applied using a hydraulic pump to simulate reservoir overburden stress, while the injection pressure was maintained below 400 psi to avoid fracturing. Core saturation was achieved through vacuum saturation with synthetic brine for 24 hours, followed by continuous brine injection at 1 mL/min for 5 pore volumes to ensure complete water saturation^70^. Crude oil was then injected at 0.5 mL/min until water production ceased, typically after 3-4 pore volumes, establishing S_wi_ conditions. All displacement experiments were conducted at a constant reservoir temperature of 70 °C using a temperature-controlled oven and an injection pressure of 400 psi. Secondary recovery was simulated through water flooding over a range of 0.5 to 14 pore volumes to determine residual oil saturation (S_or_) and monitor pressure drop behavior. Produced oil volumes were measured volumetrically, and S_or_ was calculated accordingly.

5 $$S_{or} = \frac{{\left( {V_{w} - V_{o} } \right)}}{{V_{p} }}$$

Tertiary recovery was then modeled by injecting xanthan gum solution, followed by CO_2_ flooding. To assess injection order effects, the procedure was repeated with CO_2_ injection preceding polymer flooding. The incremental oil recovered (AOR) and corresponding residual oil saturations after polymer (S_orp_) and CO_2_ injection (S_orpc_) were computed using:

6 $$S_{orp} = \frac{{\left( {V_{w} - \left( {V_{o} + V_{op} } \right)} \right)}}{{V_{p} }}$$

7 $$AOR = \left( {S_{or} - S_{orp} } \right)$$

8 $$S_{orpc} = \frac{{\left( {V_{w} - \left( {V_{o} + V_{op} } \right)} \right)}}{{V_{p} }}$$

9 $$AOR = \left( {S_{or} - S_{orpc} } \right)$$

To assess injection order effects, the procedure was repeated with CO_2_ injection preceding polymer flooding. The results were compared to identify the most effective injection sequence for maximizing oil recovery.

## Results and discussion

### Rheology and viscoelastic criteria

**Fig. 1:**
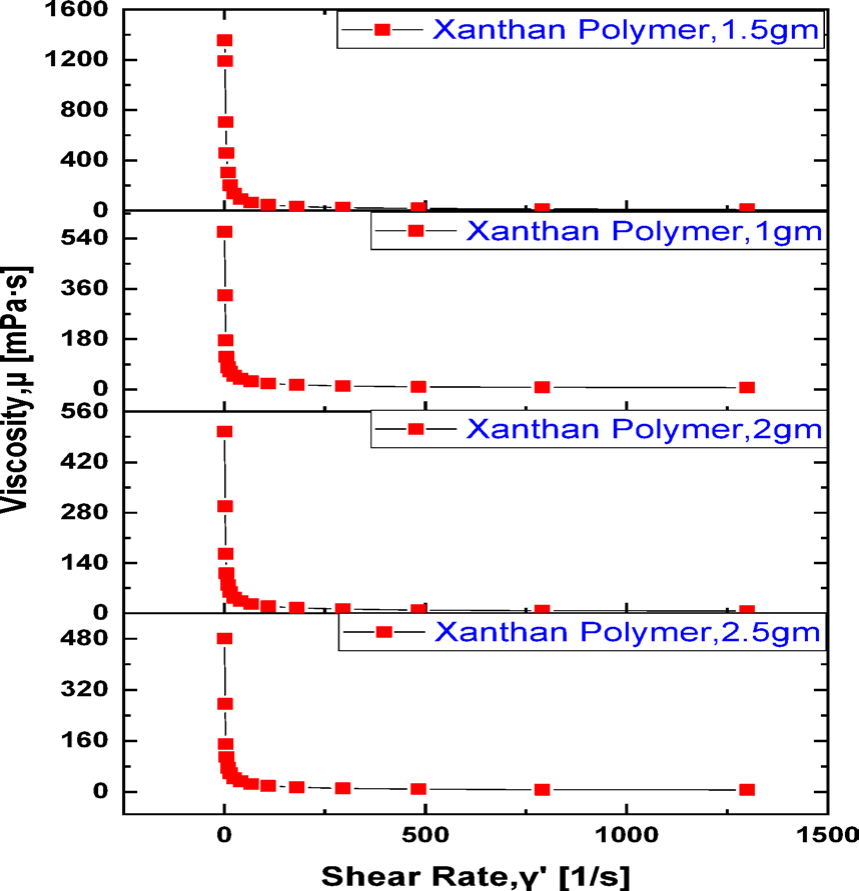
Shear rate versus viscosity for native xanthan at 70 ˚C, 135,000 ppm salinity.

**Fig. 2:**
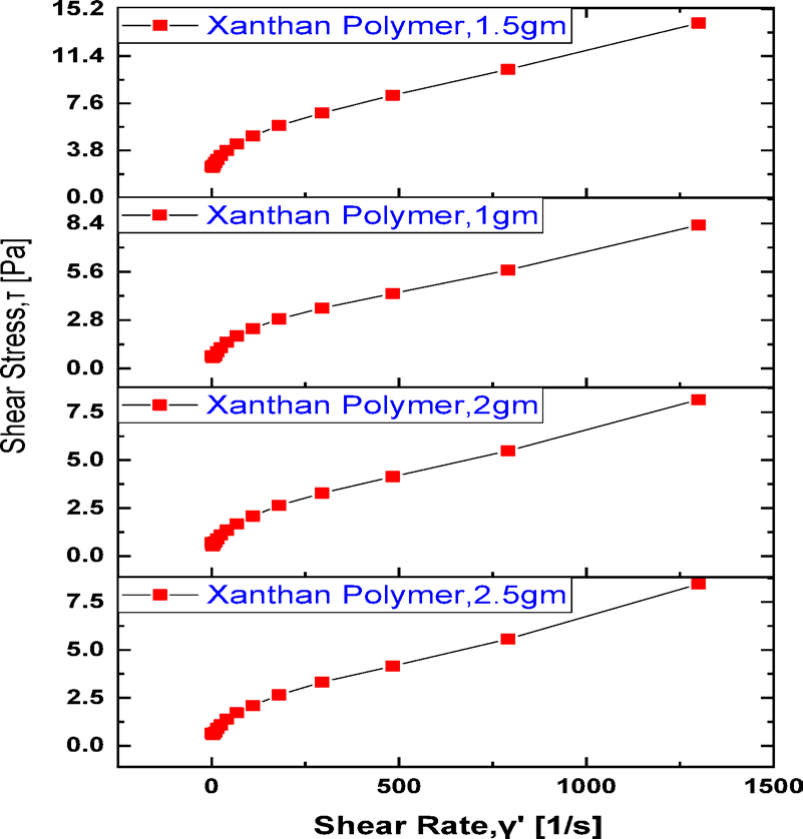
Shear rate versus shear stress for native xanthan at 70 ˚C, 135,000 ppm salinity.

The shear rate and shear stress of xanthan gum solutions were systematically investigated over a concentration range of 1.0–2.5 g/L, as illustrated in Figs. 1 and 2. Across all tested concentrations, the xanthan gum solutions exhibited pronounced **shear-thinning (pseudoplastic) behavior**, characterized by a continuous decrease in apparent viscosity with increasing shear rate. This non-Newtonian response is a well-known characteristic of xanthan-based biopolymers and is particularly advantageous for enhanced oil recovery (EOR) applications, as it facilitates injectivity at high shear rates near the wellbore while maintaining sufficient viscosity under low-shear reservoir conditions^69,71^. At low shear rates, xanthan gum molecules remain in a partially ordered and extended conformation, forming a weak three-dimensional network through intermolecular interactions and entanglements. This structural arrangement results in relatively high viscosity values, especially for polymer concentrations above the critical overlap threshold. As the shear rate increases, hydrodynamic forces progressively align and stretch the polymer chains in the direction of flow, disrupting intermolecular associations and reducing flow resistance. Consequently, the viscosity decreases with increasing shear rate, reflecting the breakdown of the polymer network under shear^72^. Among the tested concentrations, the xanthan gum solution at **1.5 g/L consistently exhibited the most favorable viscosity profile,** achieving comparatively high viscosity values while maintaining stable shear-thinning behavior. This observation can be attributed to an optimal balance between polymer chain overlap and molecular mobility. At this concentration, sufficient polymer–polymer interactions exist to form an effective flow-resisting network, yet the solution remains free from excessive chain entanglement or aggregation that could otherwise limit molecular extension and reduce rheological efficiency. In contrast, lower concentrations (e.g., 1.0 g/L) fall below the optimal overlap regime, resulting in weaker intermolecular interactions and lower viscosity. Conversely, higher concentrations (2.0–2.5 g/L) may experience partial chain crowding and increased sensitivity to salinity and temperature effects, which can suppress effective chain extension and diminish the incremental viscosity gain relative to polymer dosage^73–75^.

The rheological performance of xanthan gum was also influenced by reservoir-relevant temperature and salinity conditions. Elevated temperatures reduced viscosity due to increased molecular mobility and free volume within the polymer structure, which shortens relaxation times and weakens intermolecular interactions. Similarly, high brine salinity adversely affected viscosity by screening the electrostatic repulsion along the xanthan backbone^75,76^. Rheological measurements were conducted at reservoir temperature (70 °C) using an Anton Paar RheoCompass™ MCR 102e rheometer equipped with a concentric cylinder geometry (CC27) and Peltier temperature control system (± 0.1 °C accuracy). All polymer solutions were prepared in synthetic brine (135,000 ppm total salinity: 80,000 ppm NaCl, 35,000 ppm CaCl_2_, 20,000 ppm MgCl_2_) to represent formation water conditions. Before measurement, samples were equilibrated at the test temperature for 10 min. A solvent trap was used to minimize evaporation during high-temperature measurements. The presence of divalent and monovalent cations neutralizes the polymer’s charged groups, promoting a conformational transition from an extended coil to a more compact helical structure. This conformational change reduces the hydrodynamic volume of the polymer molecules and limits their ability to resist flow, resulting in viscosity loss. The shear-rate-dependent rheological analysis demonstrates that xanthan gum solutions retain strong shear-thinning characteristics across the tested concentration range, with **1.5 g/L emerging as the most rheologically efficient concentration** under the investigated conditions. This concentration provides an optimal compromise between viscosity enhancement, shear stability, and resistance to thermal and salinity-induced degradation, making it particularly suitable for polymer-assisted CO_2_ flooding applications^77^.

Polymer concentration is one of the most influential parameters governing the rheological performance of xanthan gum solutions and, consequently, their effectiveness in enhanced oil recovery (EOR) processes. From Fig. 1, a general increase in viscosity was observed as the polymer concentration increased, reflecting the progressive development of intermolecular interactions and polymer chain overlap within the aqueous phase. At low polymer concentration (1.0 g/L), the xanthan gum molecules are relatively isolated, with limited chain overlap and weak intermolecular entanglement. Under these conditions, the solution exhibits modest viscosity enhancement, and the polymer network formed is insufficient to provide strong resistance to flow, particularly under reservoir shear conditions. As a result, mobility control at this concentration is limited, reducing its effectiveness for improving sweep efficiency during flooding operations^78^. Increasing the concentration to **1.5 g/L** leads to a marked increase in viscosity, indicating the onset of a semi-entangled polymer regime. At this concentration, the xanthan gum chains begin to overlap and interact more extensively, forming a more coherent and elastic network capable of resisting flow deformation. This enhanced molecular interaction significantly improves the solution’s apparent viscosity, especially at low-to-moderate shear rates, which are representative of in-situ reservoir flow conditions. Importantly, the 1.5 g/L solution maintains stable shear-thinning behavior, allowing high injectivity near the wellbore while preserving sufficient viscosity deeper in the reservoir^79^. Further increases in polymer concentration to 2.0 and 2.5 g/L result in additional viscosity enhancement; however, the incremental gains become progressively less pronounced. This diminishing return can be attributed to molecular crowding and increased chain entanglement, which restrict the effective extension of polymer chains under flow. At higher concentrations, the solution becomes more susceptible to thermal degradation and salinity-induced charge screening, particularly in high-salinity brines, which can suppress the expected viscosity increase. Moreover, excessive polymer loading may lead to operational challenges such as increased injection pressure, reduced injectivity, and higher chemical costs without proportional improvement in displacement efficiency^80^. From a flow behavior perspective, all concentrations exhibit non-Newtonian pseudoplastic characteristics; however, the degree of shear-thinning becomes more pronounced as polymer concentration increases. The 1.5 g/L solution demonstrates an optimal balance between viscosity enhancement and flow adaptability, providing sufficient resistance to viscous fingering while avoiding excessive pressure buildup. This balance is particularly advantageous for hybrid polymer–CO_2_ flooding, where controlled mobility and stable displacement fronts are critical for delaying gas breakthrough and maximizing sweep efficiency^81^. Increasing xanthan gum concentration enhances viscosity and improves flow control. The results indicate that **1.5 g/L represents an optimal concentration** under the investigated conditions. This concentration achieves high and stable viscosity, favorable shear-thinning behavior, and improved tolerance to reservoir salinity and temperature, making it a technically and economically efficient choice for polymer-assisted CO_2_ flooding applications.

Figure 2 presents the relationship between shear stress and shear rate for xanthan gum solutions at different concentrations. The results indicate a nonlinear increase in shear stress with increasing shear rate, confirming the non-Newtonian, shear-thinning nature of the biopolymer solutions. This deviation from linearity reflects the progressive structural changes occurring within the polymer network as the applied shear rate increases^82^. At low shear rates, xanthan gum molecules exist in a highly entangled and partially crosslinked configuration, forming a transient network that offers strong resistance to flow. Under these conditions, relatively high shear stress is required to initiate deformation, as the polymer chains are randomly oriented and interact through intermolecular associations. As the shear rate increases, hydrodynamic forces overcome these interactions, leading to gradual disentanglement and alignment of the polymer chains in the flow direction. This molecular reorientation reduces the number of effective crosslinking points and weakens intermolecular resistance, resulting in a lower rate of increase in shear stress relative to shear rate. The observed reduction in flow resistance at higher shear rates is particularly beneficial for polymer flooding operations^74,83^. As polymer solutions are injected into porous media, they experience elevated shear rates in constricted pore throats and near-wellbore regions. The shear-thinning response allows the xanthan gum solution to flow more easily through narrow pore channels with reduced pressure loss and minimized mechanical degradation of the polymer chains. This behavior enhances injectivity and preserves polymer integrity, which is critical for maintaining viscosity and mobility control deeper within the reservoir where shear rates are lower^84^. The shear stress–shear rate behavior demonstrates that xanthan gum solutions adapt dynamically to changing flow conditions, combining high resistance under low-shear reservoir conditions with reduced flow resistance under high-shear injection conditions. This rheological adaptability supports efficient displacement, minimizes polymer rupture, and improves the overall effectiveness of polymer-assisted enhanced oil recovery processes.

**Fig. 3:**
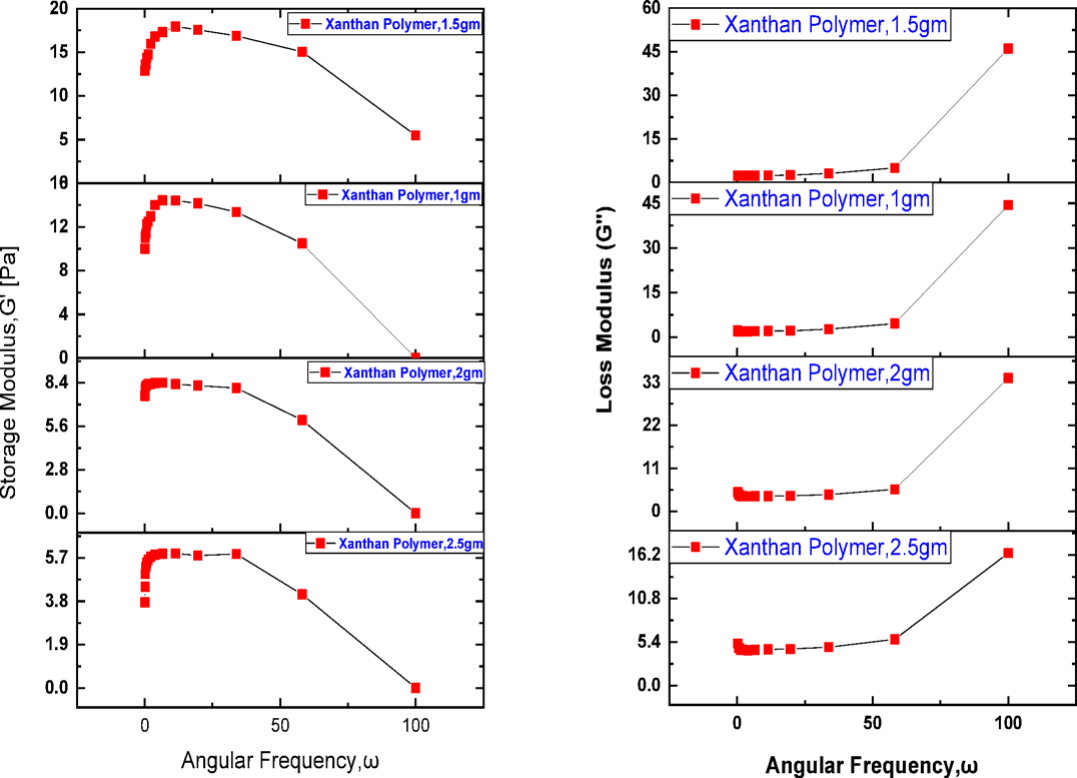
Storage/ loss modulus versus angular frequency for xanthan.

The viscoelastic behavior of xanthan gum solutions was evaluated using frequency sweep measurements, as illustrated in Fig. 3, where the angular frequency is plotted against the storage modulus (G′) and loss modulus (G″) on a logarithmic scale for polymer concentrations ranging from 1.0 to 2.5 g/L. For all tested concentrations, both moduli exhibit a linear dependence on angular frequency in the log–log domain, indicating a stable viscoelastic response over the investigated frequency range. Across all concentrations, the storage modulus consistently exceeds the loss modulus (G′ > G″), demonstrating that the elastic component dominates the viscous response. This behavior reflects the ability of xanthan gum macromolecules to form a semi-structured network capable of storing deformation energy rather than dissipating it entirely as viscous flow. The dominance of G′ suggests a high molecular weight distribution and strong intermolecular interactions, which are essential for effective mobility control during polymer flooding^85–87^.

The ratio of G″/G′ remains greater than 0.2 for all samples, classifying the system as a weak gel. Such a mechanical spectrum is characteristic of physically entangled polymer networks that retain elastic features while remaining sufficiently deformable to flow through porous media. Notably, the storage modulus values exceed 10 Pa at optimal concentrations, indicating a transition toward strong gel-like behavior^14^. Materials exhibiting this level of elasticity possess enhanced energy storage capability and a greater tendency to recover their original structure after the removal of applied stress. This viscoelastic response plays a crucial role in enhanced oil recovery applications. A higher storage modulus enhances the resistance of the displacing fluid to extensional and shear deformation, allowing the polymer solution to maintain its integrity within the reservoir^88^. This elastic contribution improves microscopic displacement efficiency by enabling the polymer to pull and mobilize trapped oil droplets from pore constrictions and dead-end pores, thereby reducing residual oil saturation. The statement “ratio > 0.2 indicates mobility reduction” requires clearer interpretation. A condition where G″/G′ > 0.2 (i.e., tan δ > 0.2) while G′ remains greater than G″ reflects a weak gel-like behavior, in which elastic effects dominate but viscous dissipation is still present. This viscoelastic response is closely linked to improved mobility control in porous media. The elastic component, in particular, enhances flow resistance within pore constrictions, mitigates viscous fingering, and promotes a more uniform displacement front, ultimately leading to improved sweep efficiency^89^. In porous media flow, this elasticity generates normal stresses that assist in mobilizing trapped oil and improving displacement efficiency. Consequently, polymer solutions with higher elastic contributions can effectively reduce the mobility of injected gas and stabilize the displacement front.”

In addition, elastic-dominant behavior contributes to improved macroscopic sweep efficiency by stabilizing the displacement front and mitigating viscous fingering. Temperature effects further influence the viscoelastic performance of xanthan gum solutions. A reduction in storage modulus with increasing temperature indicates a weakening of intermolecular forces within the polymer network, resulting in a gradual shift from elastic-dominated flow toward more plastic or viscous behavior^90,91^. This transition reflects increased molecular mobility and reduced chain interactions at elevated temperatures, which may impact long-term mobility control if not properly accounted for in polymer design. The combined storage and loss modulus behavior confirms that xanthan gum solutions behave as viscoelastic solids at higher frequencies, where G′ remains greater than G″. This rheological signature highlights the suitability of xanthan gum for polymer flooding applications, as its elastic dominance enhances injectivity control, improves sweep efficiency, and supports effective CO_2_ mobility reduction when applied in hybrid EOR processes^92^.

### Viscoelastic properties of xanthan gum and their impact on co_2_ mobility control

The viscoelastic behavior of xanthan gum solutions, evaluated through frequency sweep tests, provides critical insight into their effectiveness for mobility control in combined polymer–CO_2_ enhanced oil recovery processes. The storage modulus (G′) and loss modulus (G″) were measured over a wide angular frequency range for polymer concentrations between 1.0 and 2.5 g/L. For all tested solutions, both moduli exhibited a linear relationship with angular frequency on a logarithmic scale, indicating a stable and predictable viscoelastic response under dynamic flow conditions. In all cases, the storage modulus consistently exceeded the loss modulus (G′ > G″), confirming that the elastic component dominates the viscous response of the xanthan gum solutions. This elastic dominance reflects the presence of a physically entangled polymer network capable of storing deformation energy during flow^78^. Such behavior is particularly advantageous in porous media, where elastic forces assist in mobilizing trapped oil by generating normal stresses that act perpendicular to the flow direction. These elastic stresses enhance microscopic displacement efficiency by pulling oil droplets out of pore throats and dead-end pores, a mechanism not achievable through purely viscous flow^93^.

The ratio of G″/G′ remained greater than 0.2 across all concentrations, indicating that the polymer systems can be classified as weak gels. Weak gel behavior is highly desirable for EOR applications because it combines sufficient elasticity for mobility control with adequate deformability to ensure injectivity^94^. At polymer concentrations of 1.5 g/L and above, the storage modulus exceeded 10 Pa, suggesting a transition toward stronger gel-like behavior. Higher G′ values correspond to increased energy storage capacity and improved structural recovery after deformation, enabling the polymer to maintain its rheological integrity during repeated shearing within the reservoir^95^. The viscoelastic contribution of xanthan gum plays a decisive role in controlling CO_2_ mobility during combined flooding operations. CO_2_ injection alone often suffers from early breakthrough and severe channeling due to its low viscosity and high mobility^10,96^. When preceded or accompanied by a viscoelastic polymer slug, the elastic resistance of the polymer solution reduces gas mobility, stabilizes the displacement front, and improves macroscopic sweep efficiency. The elastic nature of xanthan gum also enhances resistance to extensional flow at pore constrictions, thereby mitigating CO_2_ fingering and promoting a more uniform saturation profile^92^. Among the tested concentrations, 1.5 g/L exhibited the most favorable balance between viscosity enhancement, elastic strength, and flowability. At this concentration, the polymer solution achieved high viscosity and a pronounced elastic response without excessive molecular entanglement or flow resistance. Lower concentrations (1.0 g/L) exhibited insufficient elasticity and reduced mobility control, while higher concentrations (2.0–2.5 g/L) showed diminishing incremental gains in G′, accompanied by increased risk of injectivity impairment and mechanical degradation under high shear. The superior performance of the 1.5 g/L solution can therefore be attributed to an optimal polymer network structure, where chain entanglement and molecular interactions are sufficient to generate strong viscoelastic effects while preserving stable flow through porous media. The viscoelastic characterization confirms that xanthan gum solutions behave as viscoelastic solids over a wide frequency range, with elastic dominance (G′ > G″) that is essential for effective mobility control. The optimal concentration of 1.5 g/L provides a synergistic balance between elasticity, viscosity, and injectivity, making it particularly effective for reducing CO_2_ mobility, delaying gas breakthrough, and maximizing oil recovery in hybrid polymer-CO_2_ flooding schemes.

### Sandpack flooding tests

**Fig. 4:**
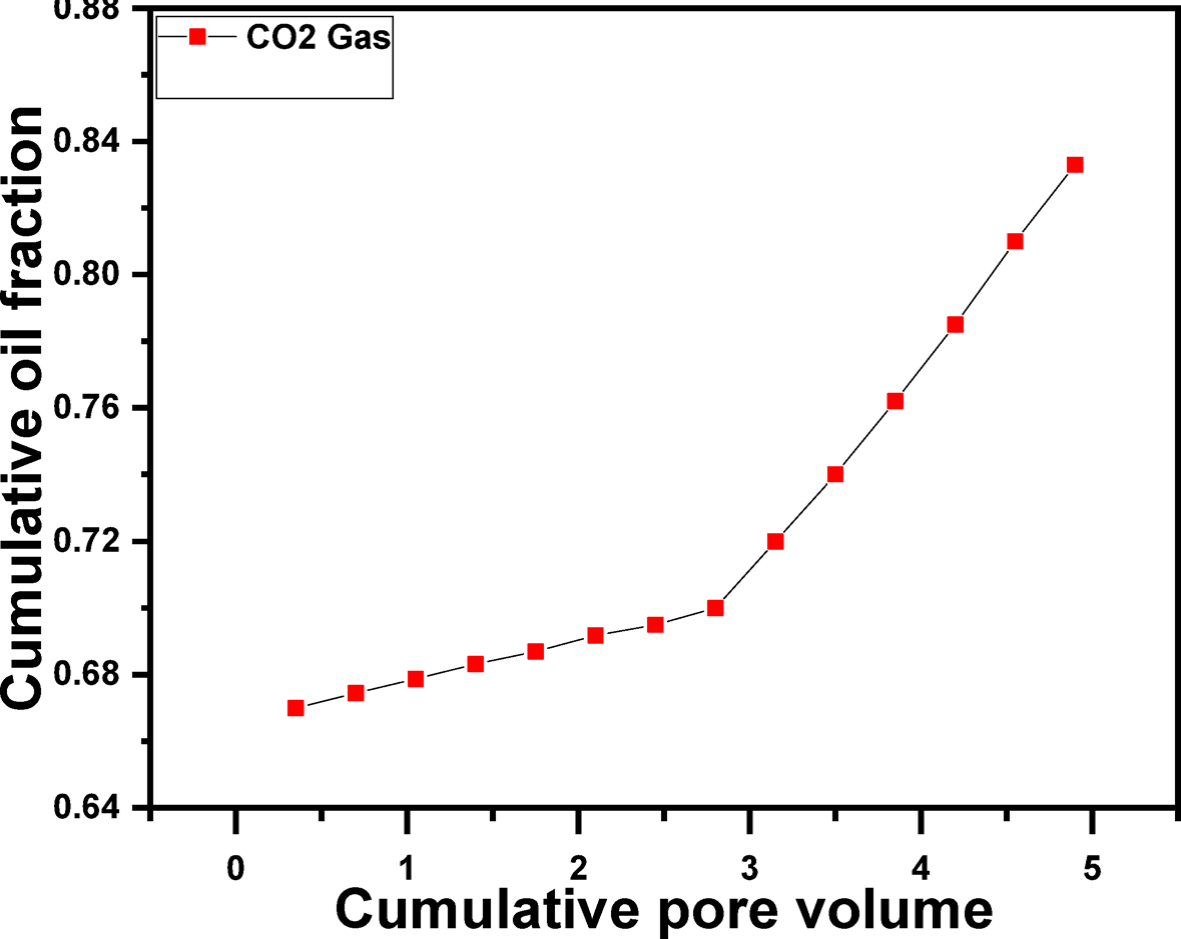
Recovered oil by CO_2_ flooding.

**Fig. 5:**
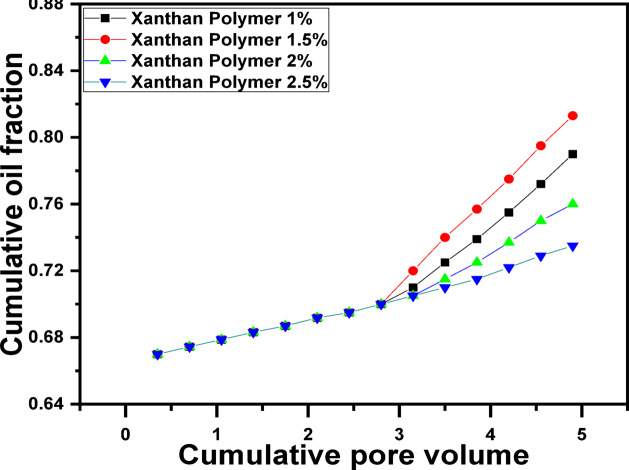
Recovered oil by different concentrations of Xanthan gum.

**Fig. 6:**
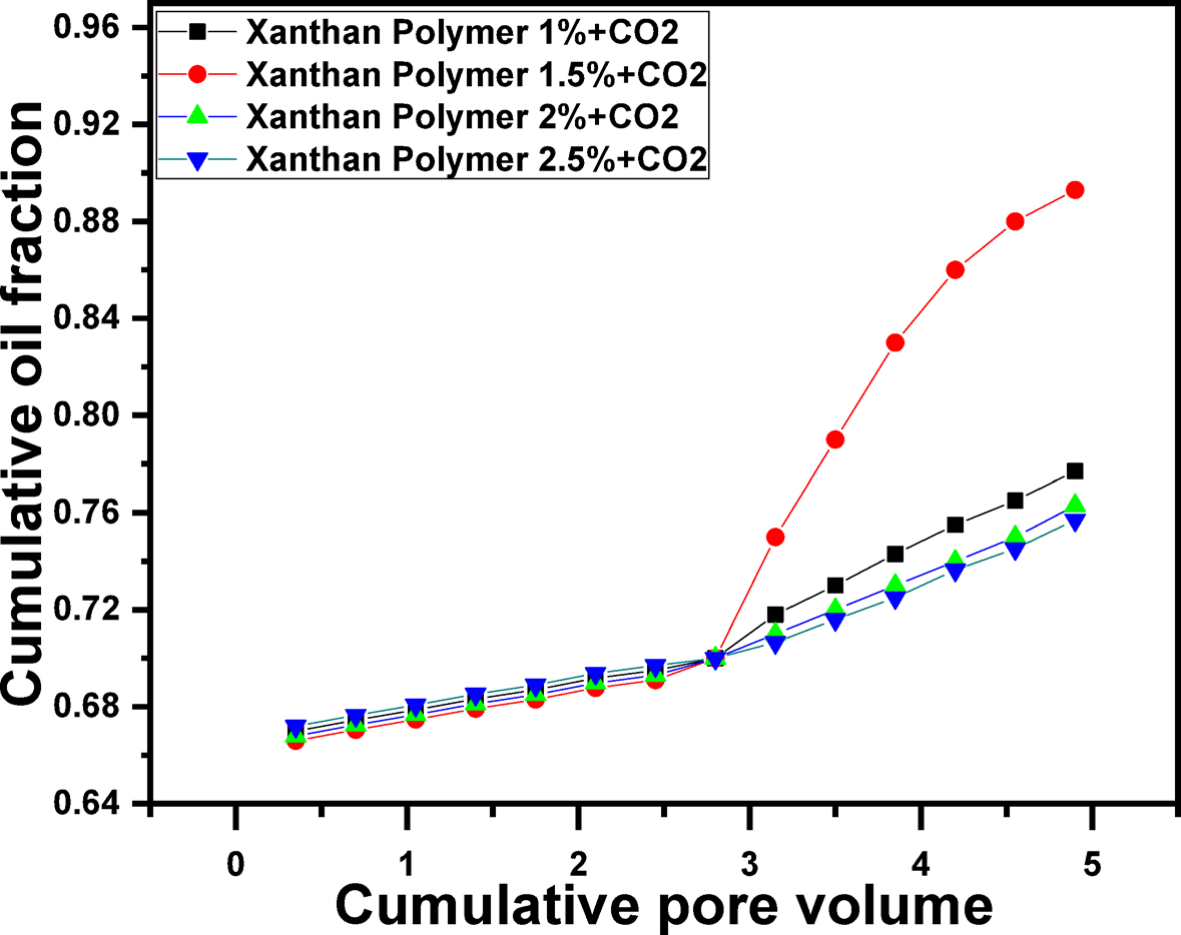
Recovered oil by different Xanthan gum concentrations followed by CO_2_ Gas flooding.

**Fig. 7:**
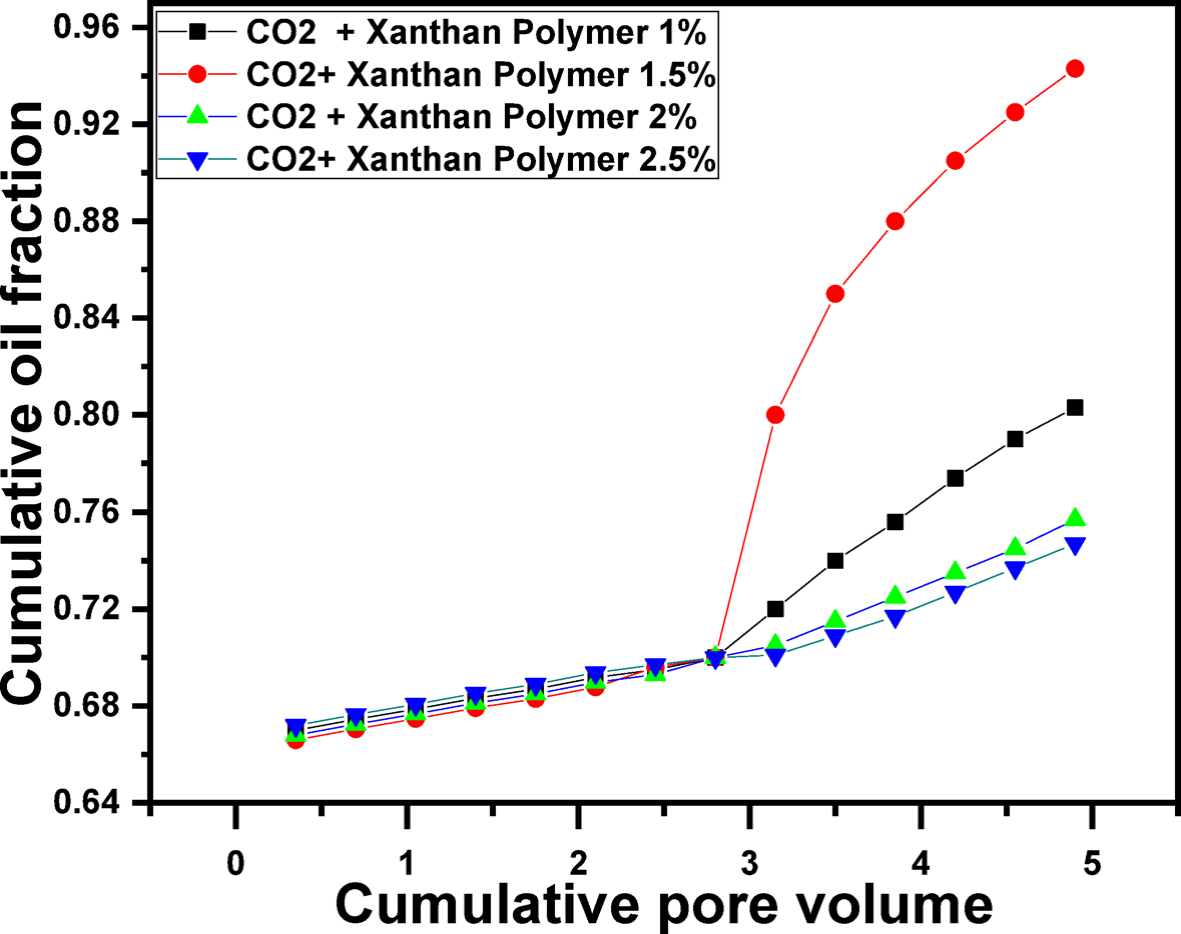
Recovered oil by CO_2_ Gas followed by Xanthan Polymer with different concentrations.

**Fig. 8:**
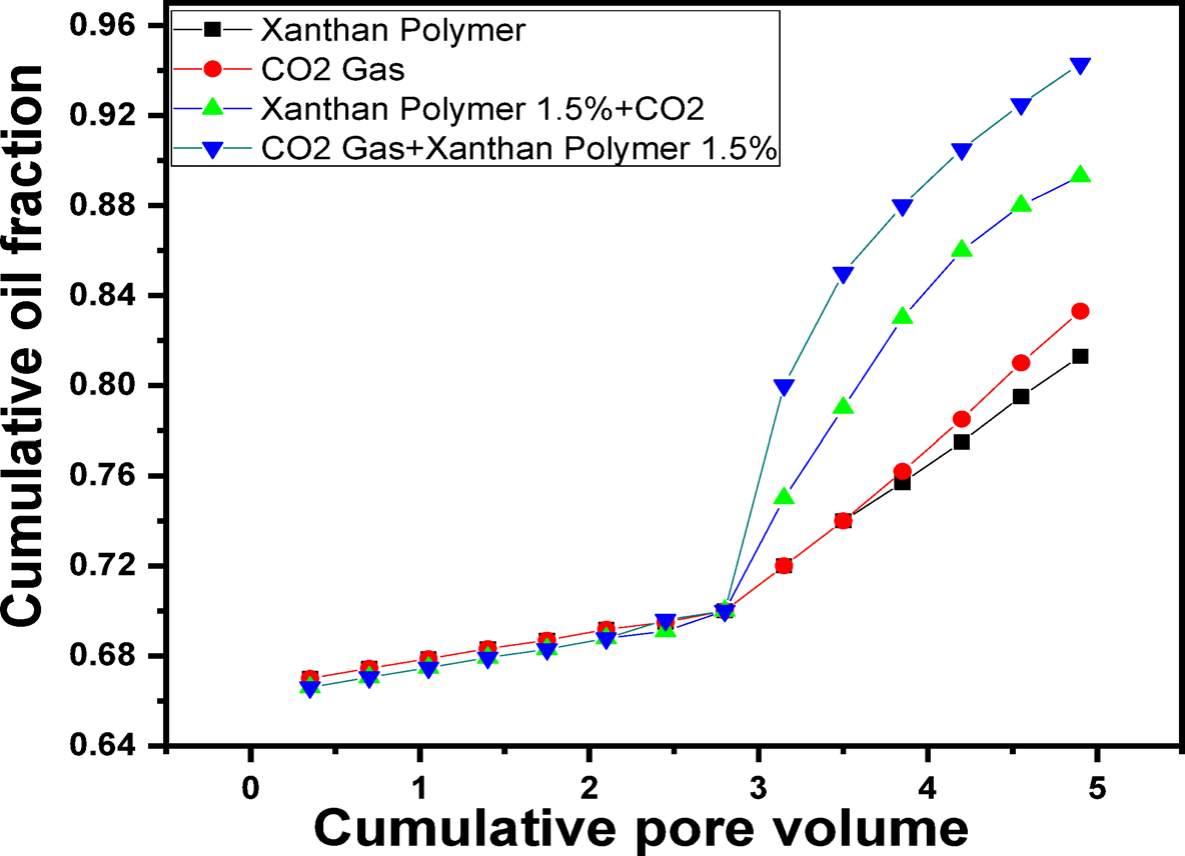
maximum oi Recovery in the four scenarios.

A series of flooding experiments was conducted using homogeneous sandstone sand-pack models to evaluate the performance of water flooding, CO_2_ flooding, polymer flooding, and hybrid polymer-CO_2_ flooding schemes. Each flooding scenario was performed on a freshly packed model with nearly identical petrophysical properties, including porosity and permeability, to ensure consistency and eliminate the influence of rock heterogeneity on recovery trends. The flooding sequences and corresponding recovery factors are presented in Figs. 4–8.

#### Water flooding (baseline case)

Water flooding was initially conducted in all experiments to establish a baseline recovery and simulate secondary oil recovery. Water injection continued until oil production ceased, resulting in an ultimate recovery of approximately 70% of the original oil in place (OOIP). This recovery level is typical for homogeneous sandstone systems under favorable mobility conditions. However, the remaining 30% OOIP represents bypassed and capillary-trapped oil, primarily caused by unfavorable mobility ratios, viscous fingering, and incomplete sweep efficiency, highlighting the necessity for tertiary recovery methods^33^.

#### CO_2_ flooding alone

Following the baseline water flooding case, CO_2_ flooding was evaluated as a standalone EOR method (Fig. 4). CO_2_ injection resulted in a significant improvement in oil recovery, reaching approximately 83.3% OOIP. This enhancement can be attributed to CO_2_–oil interactions, including oil swelling, viscosity reduction, interfacial tension (IFT) lowering, and partial miscibility under the applied experimental conditions. Despite this improvement, CO_2_ flooding alone is still limited by early gas breakthrough and gravity override, which reduce sweep efficiency and leave portions of the reservoir unswept^97^.

#### Xanthan gum polymer flooding

Polymer flooding experiments were conducted using xanthan gum concentrations ranging from 1.0 to 2.5 g/L to evaluate the effect of polymer concentration on oil recovery (Fig. 5). The results demonstrate that polymer injection significantly improves recovery compared with water flooding due to enhanced mobility control and improved sweep efficiency. Among the tested concentrations, the 1.5 g/L xanthan gum solution achieved the highest recovery, reaching approximately 81.3% OOIP. At lower concentrations (1.0 g/L), the polymer viscosity and elastic strength were insufficient to effectively correct the mobility ratio, resulting in limited incremental recovery^98^. Conversely, higher concentrations (2.0–2.5 g/L) did not yield proportional increases in oil recovery. This behavior can be attributed to excessive polymer chain entanglement, which increases flow resistance, reduces injectivity, and limits effective propagation through the porous medium^99^. The superior performance of the 1.5 g/L solution reflects an optimal balance between viscosity enhancement, viscoelastic response, and flowability.

#### Polymer flooding followed by CO_2_ injection

To investigate the combined effect of polymer and CO_2_ flooding, experiments were conducted in which xanthan gum solutions were injected first, followed by CO_2_ flooding (Fig. 6). This hybrid strategy resulted in a notable increase in oil recovery, with the 1.5 g/L xanthan gum case achieving approximately 89.3% OOIP. In this sequence, the polymer slug improves mobility control and establishes a more uniform displacement front, reducing permeability contrasts and mitigating channeling pathways. Subsequent CO_2_ injection benefits from this improved sweep efficiency, allowing better contact between CO_2_ and the remaining oil. The polymer slug also reduces CO_2_ mobility, delaying gas breakthrough and enhancing CO_2_ utilization efficiency^100^. Once again, the 1.5 g/L concentration demonstrated the most effective performance due to its optimal rheological and viscoelastic properties.

#### CO_2_ Flooding followed by polymer injection

The final flooding scenario reversed the injection order, initiating flooding with CO_2_ followed by xanthan gum solutions of varying concentrations (Fig. 7). This sequence produced the highest oil recovery among all tested scenarios, with a maximum recovery of approximately 94.3% OOIP achieved in the case of CO_2_, followed by 1.5 g/L xanthan gum. The superior performance of this sequence highlights the strong synergistic interaction between CO_2_ and polymer flooding. Initial CO_2_ injection mobilizes oil by reducing viscosity, swelling the oil phase, and lowering IFT, thereby displacing oil from smaller pore spaces. However, an early CO_2_ breakthrough can still occur. The subsequent polymer injection effectively blocks high-permeability flow paths, redistributes the flow toward previously unswept zones, and stabilizes the displacement front. This combination maximizes both microscopic displacement efficiency (from CO_2_) and macroscopic sweep efficiency (from polymer flooding), leading to the highest overall recovery^101^.

#### Comparative analysis and optimal flooding strategy

Figure 8 summarizes the maximum oil recovery achieved under each flooding scenario. The results clearly indicate that hybrid polymer–CO_2_ flooding outperforms single-method EOR approaches. Among all tested cases, CO_2_ flooding followed by 1.5 g/L xanthan gum injection yielded the highest recovery factor, confirming this sequence as the most effective strategy. The consistent superiority of the 1.5 g/L xanthan gum concentration across all hybrid flooding scenarios underscores its optimal rheological and viscoelastic characteristics. At this concentration, xanthan gum provides sufficient viscosity and elasticity to control mobility and improve sweep efficiency without compromising injectivity or causing excessive flow resistance^102^. These findings demonstrate that both polymer concentration and injection sequence play critical roles in maximizing EOR performance. The flooding test results confirm that combining xanthan gum polymer flooding with CO_2_ injection significantly enhances oil recovery compared with individual methods. The synergistic interaction between polymer mobility control and CO_2_ displacement mechanisms offers a robust and efficient EOR strategy for mature sandstone reservoirs. The flooding results for each scenario are summarized in Table 1.

**Table 1:** Summary of flooding results for each scenario.

Flooding Scenario	Oil Recovery (% OOIP)
Water flooding (baseline)	70.0
CO_2_ flooding alone	83.3
Polymer flooding (1.0 g/L)	~ 79
Polymer flooding (1.5 g/L)	81.3
Polymer flooding (2.0 g/L)	~ 76
Polymer flooding (2.5 g/L)	~ 73.5
Polymer (1.5 g/L) → CO_2_	89.3
CO_2_ → Polymer (1.5 g/L)	94.3

## Numerical model description

**Fig. 9:**
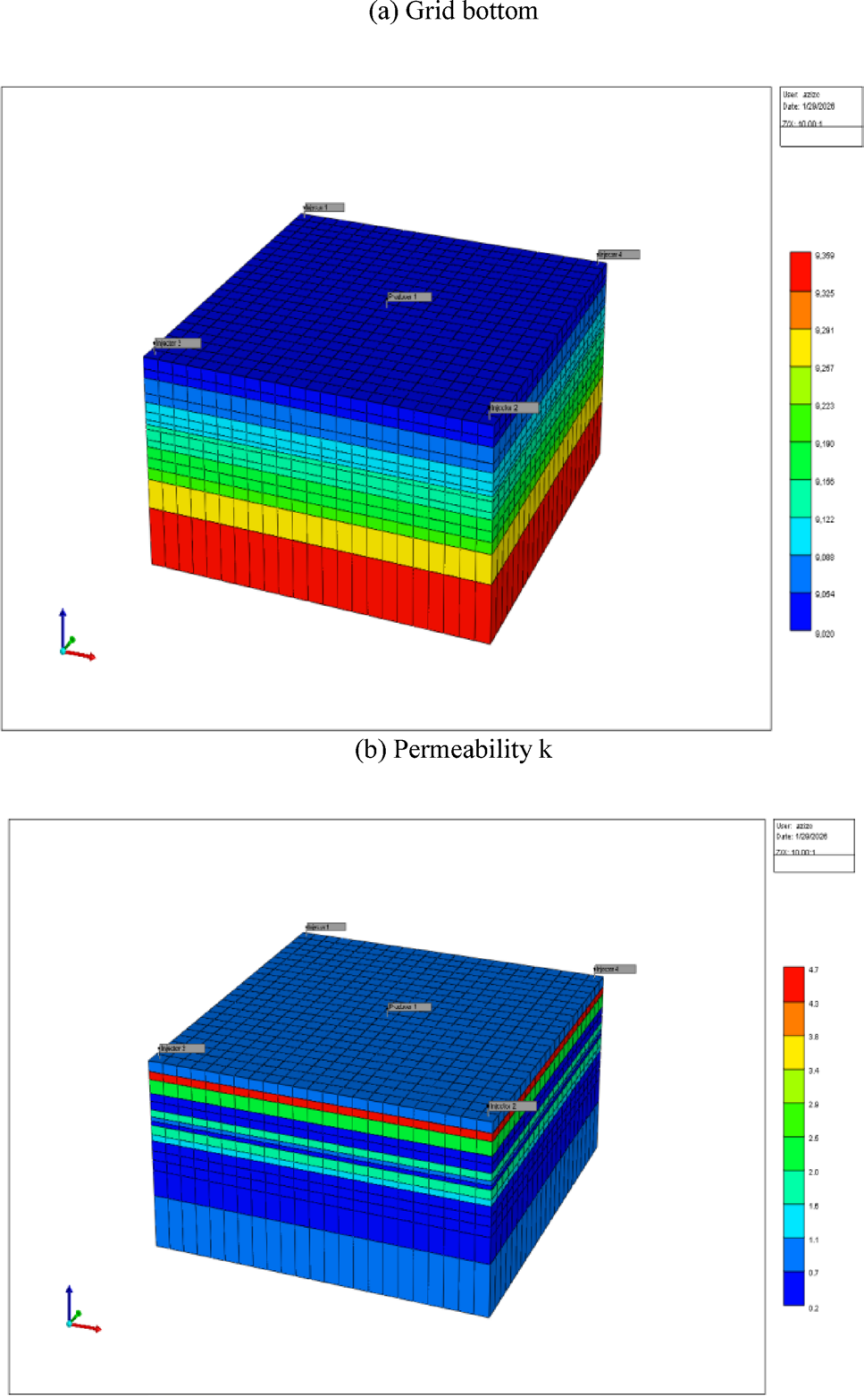

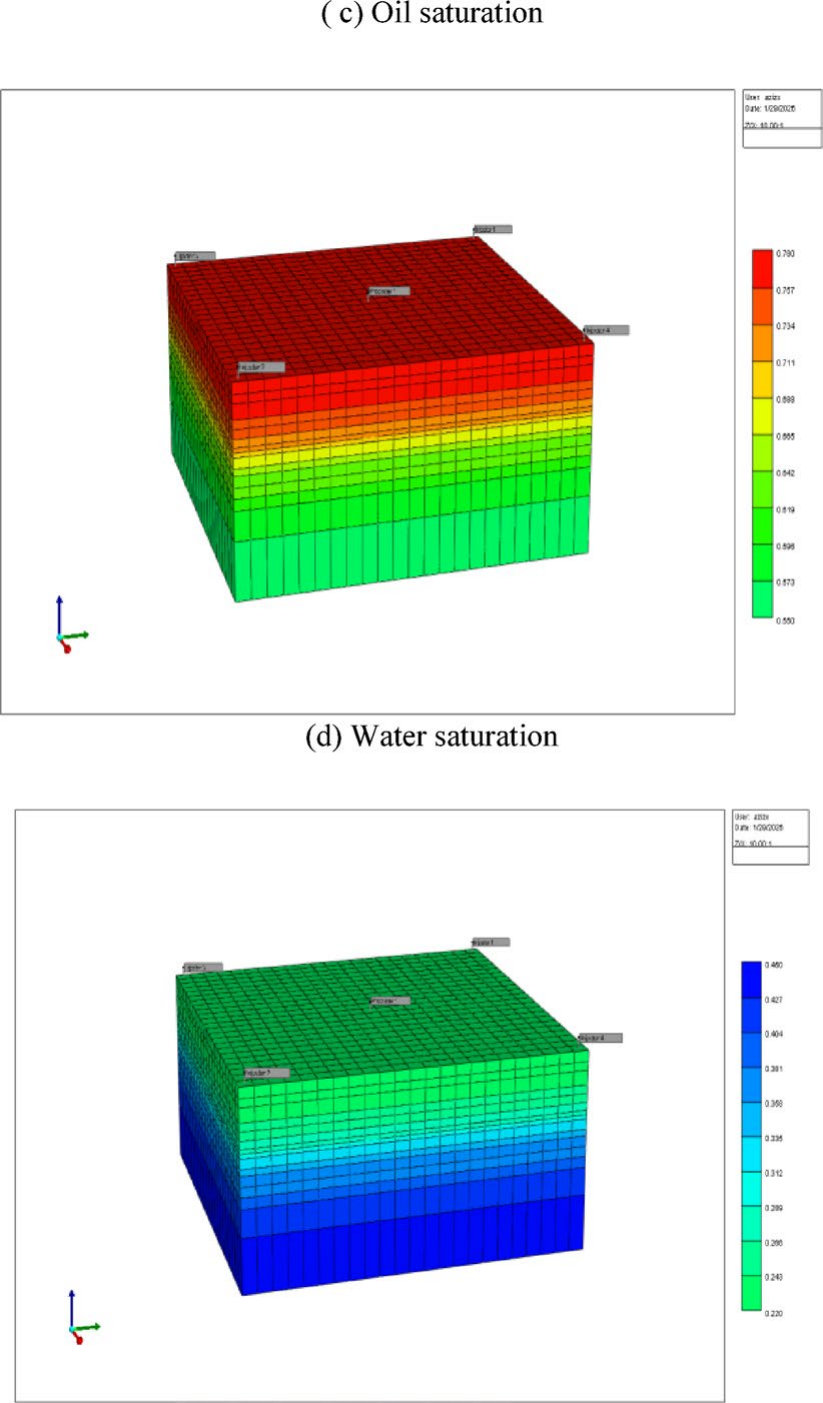
reservoir geological mosel as a function of Grid bottom, permeability (k); Oil saturation, and water saturation.

The reservoir was modeled using CMG-IMEX (black-oil simulator) to evaluate the impact of CO_2_ and xanthan gum polymer injection on oil recovery and water production performance. The simulation period extended from 1 January 2026 to 1 January 2029, allowing sufficient time to capture early breakthrough, sweep development, and medium-term EOR response. A ¼ five-spot pattern was selected to represent field-scale displacement behavior while minimizing computational cost^103^. The model consisted of four injector wells located at the corners and one central producer, a configuration widely used in waterflood and chemical EOR studies due to its symmetry and well-defined areal sweep characteristics. The reservoir geometry covered an area of 7200 ft × 7500 ft, with a total thickness of 359 ft. The reservoir top was located at a depth of 9000 ft, and the vertical discretization resulted in 15 layers with an average layer thickness of approximately 24 ft, enabling adequate resolution of vertical flow and saturation variations as shown in Fig. 9. The Cartesian grid system contained 7935 active grid blocks, with an average areal grid size of ~ 350 ft × 350 ft, ensuring numerical stability while maintaining sufficient spatial resolution. Rock compressibility was set to 1 × 10⁻⁶ psi⁻^1^, representing a moderately compressible sandstone formation. Reservoir temperature and initial pressure were fixed at 200 °F and 3600 psi, respectively, conditions that are favorable for polymer stability and near-miscible CO_2_ behavior. Only the middle reservoir layers (layers 9, 10, and 11) were perforated for both injectors and the producer, focusing flow within the most productive interval and reducing the impact of gravity override and early water breakthrough. Table 2 summarizes the input PVT data, porosity, and polymer adsorption used in the simulation model.

**Table 2:** Summary of input data in simulation model.

Input Data	Value
Porosity	22%
Polymer adsorption	Polymer adsorption in the simulation model was represented using a Langmuir isotherm with a maximum adsorption of 30 μg/g rock, based on typical values for xanthan gum on sandstone
PVT values :	
Oil density at the stock tank	31°API (0.87 g/cm^3^)
Oil viscosity at reservoir conditions	6.0 cP
Water viscosity at reservoir conditions	0.5 cP
Oil formation volume factor (Bo)	1.15 rb/STB
Solution gas-oil ratio (Rs)	250 scf/STB
Rock compressibility	1 × 10⁻⁶ psi⁻^1^

Polymer flooding was simulated in CMG-IMEX using the built-in polymer module, which incorporates key physicochemical effects. The viscosity of the polymer solution was modeled as a function of concentration using the Todd–Longstaff mixing parameter, set to 0.6. Polymer adsorption onto the rock surface was represented by a Langmuir isotherm with a maximum adsorption capacity of 30 μg/g rock. A residual resistance factor (RRF) of 2.5 was applied to account for permeability reduction due to polymer retention, while shear-thinning behavior was captured using appropriate power-law coefficients. CO_2_ injection was modeled within the black-oil framework, where dissolved CO_2_ was represented through pressure-dependent solution gas–oil ratio (Rs) and oil formation volume factor (Bo). The PVT properties were derived from laboratory measurements of crude oil–CO_2_ systems at a reservoir temperature of 200 °F, with oil swelling and viscosity reduction correlated to CO_2_ concentration. Although a compositional simulator would provide a more rigorous description of phase behavior, the use of CMG-IMEX with calibrated PVT data is sufficient to capture the dominant mechanisms governing CO_2_-based EOR in this screening-level study^104^.

**Fig. 10:**
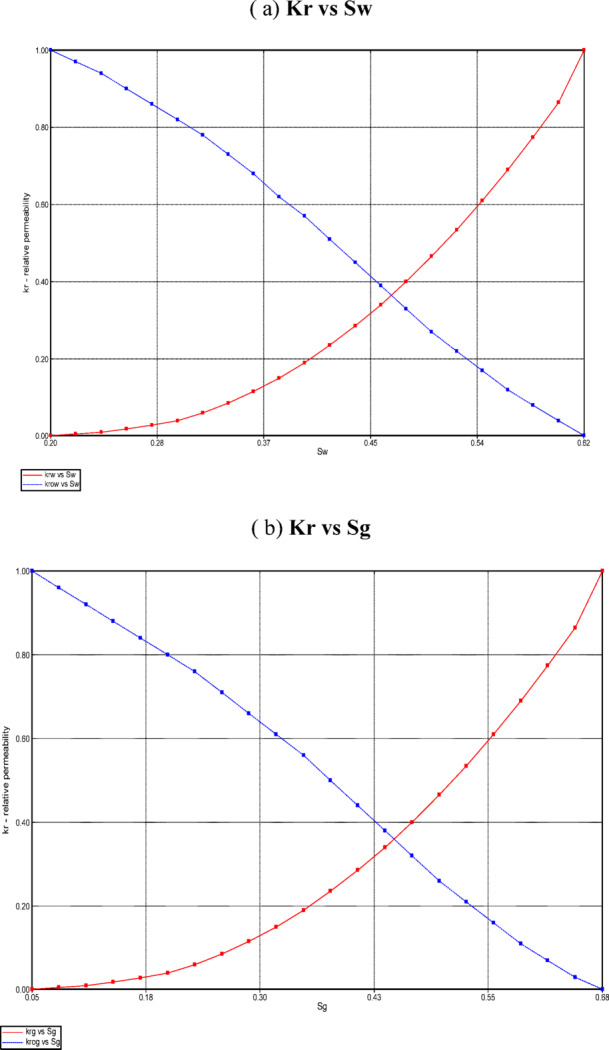
Relative permeability versus water saturation (Kr vs Sw), and gas saturation (Kr vs Sg).

The rock–fluid interaction in the reservoir is represented through relative permeability curves describing the relationships between phase permeability and fluid saturation, specifically **oil–water (Kr vs Sw)** and **oil–gas (Kr vs Sg)** systems, as displayed in Fig. 10. These curves play a critical role in controlling multiphase flow behavior, displacement efficiency, and overall recovery performance in the CMG-IMEX simulation. For the **oil–water system**, the **relative permeability versus water saturation (Kr vs Sw)** curves reflect a predominantly water-wet reservoir. At connate water saturation, oil relative permeability is at its maximum, indicating unrestricted oil flow under initial conditions. As water saturation increases during water and polymer injection, oil relative permeability gradually declines while water relative permeability increases in a smooth, monotonic manner. The gradual increase in water relative permeability and delayed decline in oil relative permeability indicate a stable displacement front and favorable mobility control. This behavior is particularly important under polymer flooding conditions, where increased water-phase viscosity further suppresses viscous fingering and improves areal and vertical sweep efficiency. The absence of curve crossing and the smooth transition between phases contribute to numerical stability and the realistic simulation of water breakthrough and post-breakthrough performance. In the **oil–gas system**, the **relative permeability versus gas saturation (Kr vs Sg)** curves describe the evolution of gas and oil flow during CO_2_ injection. Gas relative permeability remains negligible below the critical gas saturation, ensuring that gas flow does not occur prematurely. As gas saturation increases, gas relative permeability rises progressively, while oil relative permeability decreases, reflecting the increasing dominance of the gas phase in swept regions. This trend captures the essential physics of CO_2_ displacement, including improved oil mobility due to gas dissolution and partial miscibility effects. The smooth decline in oil relative permeability and the controlled increase in gas relative permeability help prevent early gas channeling and allow CO_2_ to effectively contact oil-rich zones, enhancing microscopic displacement efficiency. Together, the oil–water and oil–gas relative permeability curves ensure consistent and realistic multiphase flow behavior in the model. Their shapes support improved sweep efficiency, delayed water and gas breakthrough, and enhanced oil recovery under the combined CO_2_–polymer injection strategy. Proper calibration of these rock curves is therefore essential for accurately predicting production performance, saturation evolution, and the overall effectiveness of the enhanced oil recovery process.

### Well operating conditions and injection strategy

The injector wells were operated under constant bottom-hole pressure control of 20,000 psi, injecting water mixed with xanthan gum polymer, while the producer was constrained at 1500 psi. This large pressure differential ensured strong displacement forces and maintained injectivity throughout the simulation period. The use of xanthan gum polymer aimed to increase injected-phase viscosity, thereby reducing the mobility ratio between displacing and displaced fluids. This improves macroscopic sweep efficiency by suppressing viscous fingering and delaying water breakthrough. CO_2_ injection enhances oil recovery through a combination of microscopic and macroscopic mechanisms, including oil swelling and viscosity reduction, which improve oil mobility and flow efficiency. It also increases effective oil relative permeability and promotes partial miscibility with the oil phase under reservoir pressure conditions, leading to improved displacement efficiency. Additionally, gas-assisted gravity drainage within the perforated layers contributes to more effective vertical sweep and oil mobilization, collectively strengthening the overall recovery performance. The combined CO_2_–polymer process is therefore expected to synergistically enhance both sweep efficiency (polymer) and displacement efficiency (CO_2_).

### Simulation results

#### Oil recovery improvement

**Fig. 11:**
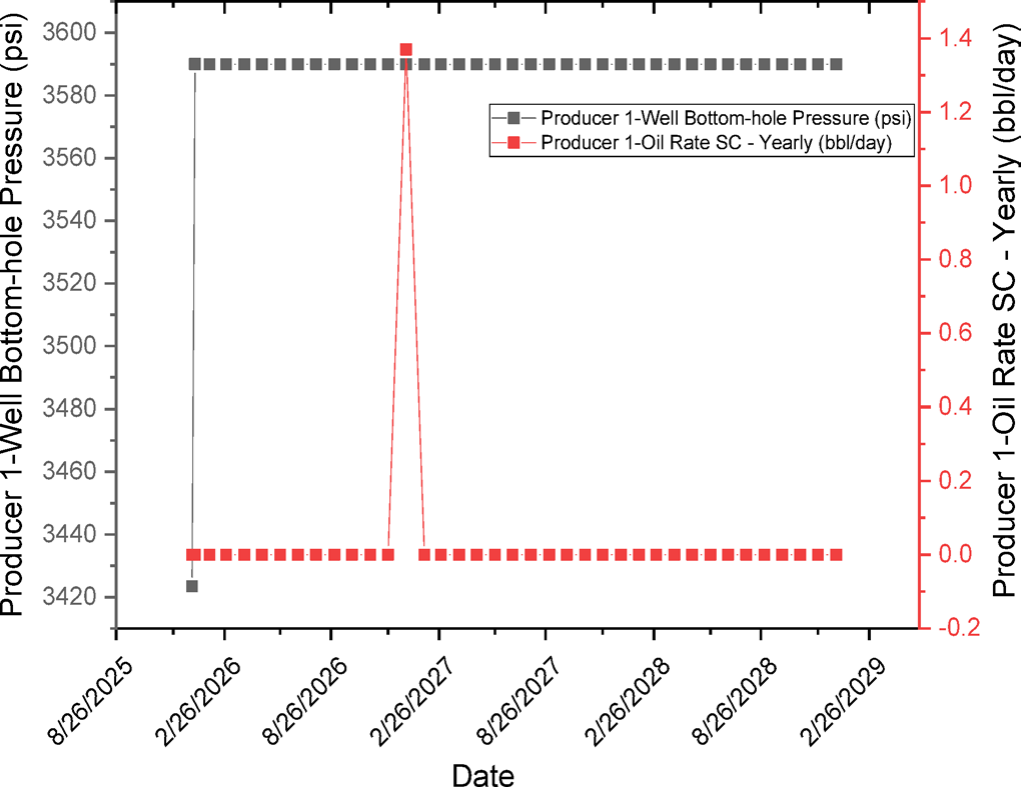
Oil production rate and well bottom- hole pressure of the producer.

Compared with conventional waterflooding, the combined CO_2_–polymer injection scenario yields higher cumulative oil production, a delayed production decline, and an improved recovery factor over the three-year simulation period. Polymer injection stabilizes the displacement front and enhances areal and vertical sweep, enabling CO_2_ to contact a larger fraction of the reservoir. The dissolution of CO_2_ into the oil phase reduces oil viscosity and increases oil mobility, resulting in incremental oil recovery beyond that achievable with polymer flooding alone. In CMG-IMEX results, these effects are reflected by increased oil relative permeability at intermediate saturations, a slower decline in oil production rates, and a higher average reservoir pressure in the vicinity of the producer, as shown in the results file (Fig. 11), supporting sustained oil production.

#### Water cut reduction and delay

**Fig. 12:**
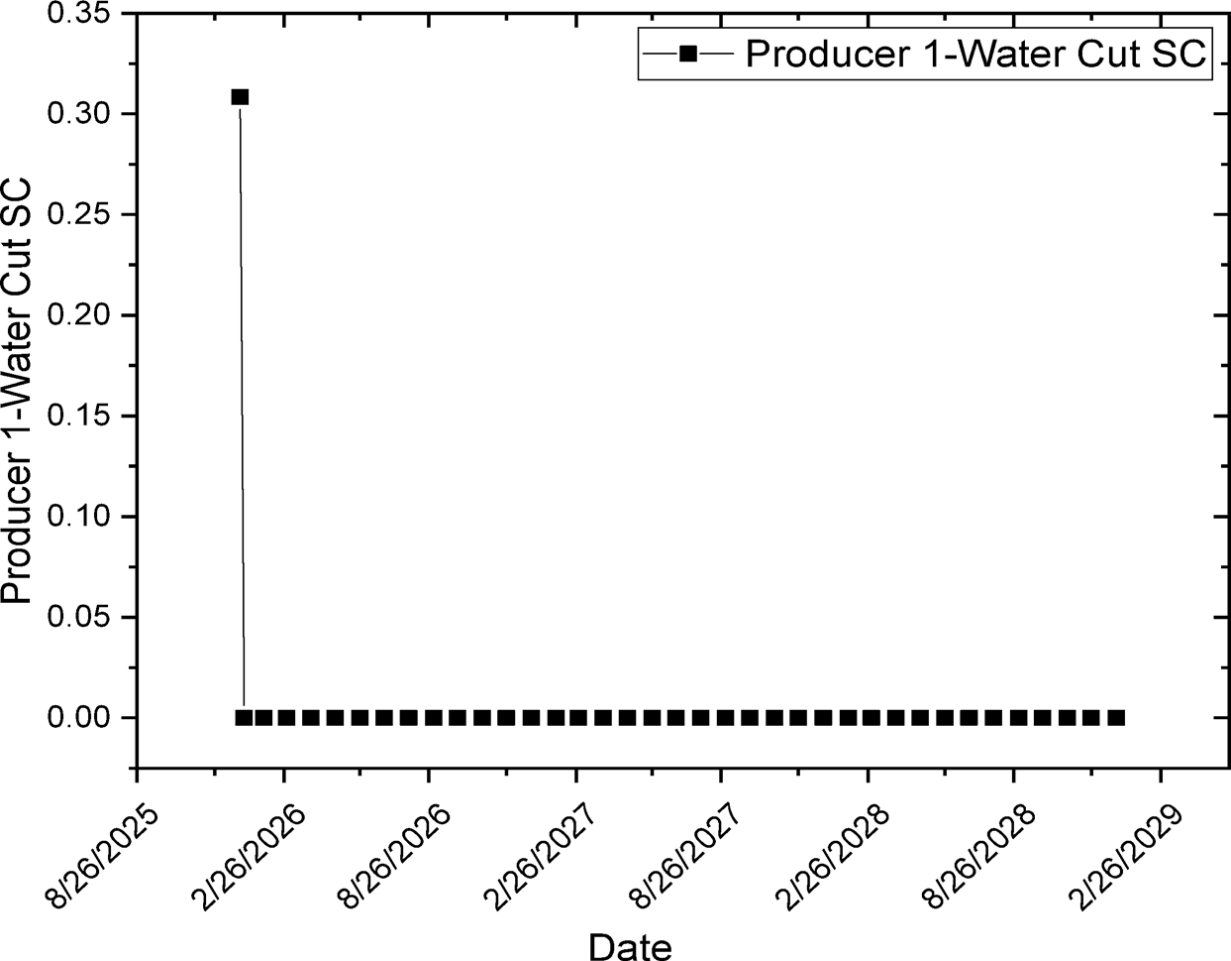
Flattened water cut curve during simulation period.

One of the most significant outcomes is reduced and delayed water cut. The polymer increases injected water viscosity, reducing channeling through high-permeability streaks and improving vertical and areal sweep. The combined CO_2_ and xanthan gum polymer injection is expected to significantly improve waterflood performance by delaying water breakthrough at the producer and reducing the peak water cut, with a more gradual increase in water production over the simulation period. In CMG-IMEX results, this behavior will be reflected by a flattened water-cut curve and a reduced water production rate, indicating improved mobility control and sweep efficiency, as shown in Fig. 12. Additionally, saturation maps will show a more uniform water and oil saturation distribution within the perforated layers, confirming enhanced areal and vertical sweep and reduced channeling of the injected fluids. This behavior is particularly enhanced by perforating only layers 9–11, which minimizes early water influx from upper and lower layers.

#### CO_2_ effects on saturation and pressure behavior

**Fig. 13:**
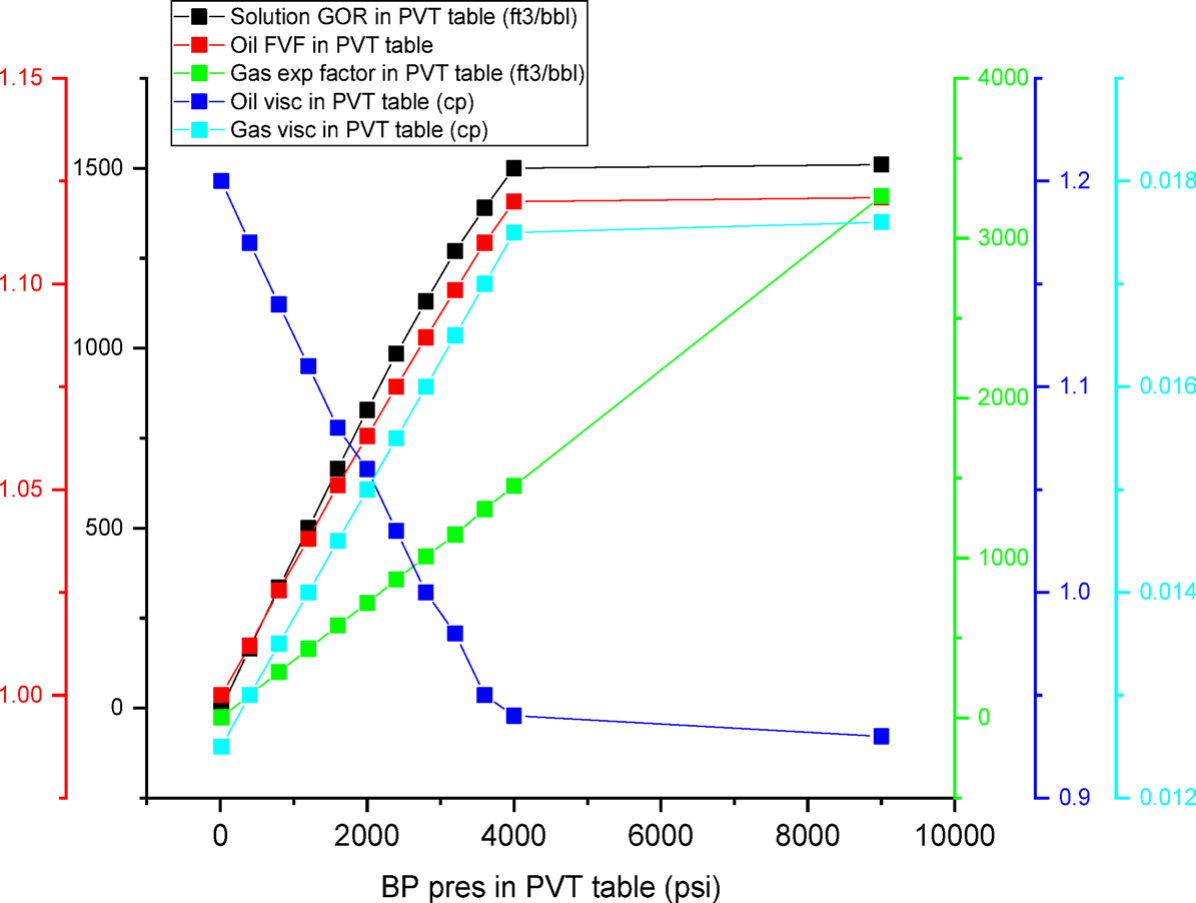
PVT properties through the simulation period.

CO_2_ injection introduces a secondary gas phase that preferentially propagates through oil-rich regions, thereby enhancing microscopic displacement efficiency. While CMG-IMEX employs a black-oil formulation rather than a fully compositional approach, it adequately captures key CO_2_-oil interactions through solution gas behavior, leading to reduced residual oil saturation and improved oil mobility. As a result, simulation outcomes show increased gas saturation in the vicinity of injection wells, lower remaining oil saturation within swept zones, and a moderate pressure buildup around injectors, which is assured with PVT properties as shown in Fig. 13, and collectively contribute to improved displacement efficiency and sustained production performance. Figure 13 illustrates the PVT data employed in the CMG-IMEX black-oil simulation, including the pressure dependence of oil formation volume factor (Bo), solution gas–oil ratio (Rs), and oil viscosity (μo), in addition to gas formation volume factor (Bg) and gas viscosity (μg). These properties were obtained from laboratory PVT analysis of a 31° API crude oil at reservoir temperature (200 °F) using a mercury-free PVT system. Standard differential liberation (DL) and constant composition expansion (CCE) tests were conducted in accordance with ASTM procedures. For CO_2_ injection, the black-oil formulation in CMG-IMEX was adapted by modifying the PVT tables to incorporate CO_2_ dissolution effects, particularly oil swelling and viscosity reduction. The PVT properties were based on standard black-oil correlations, including pressure-dependent oil formation volume factor, solution gas–oil ratio, and compressibility. Within this framework, CO_2_ behavior was represented through gas-phase properties and solution–gas interactions rather than full compositional modeling. The swelling factor and viscosity reduction were correlated to CO_2_ concentration using established correlations^105^ and further validated against experimental data from CO_2_–crude oil interaction tests.

#### Areal and vertical sweep efficiency

**Fig. 14:**
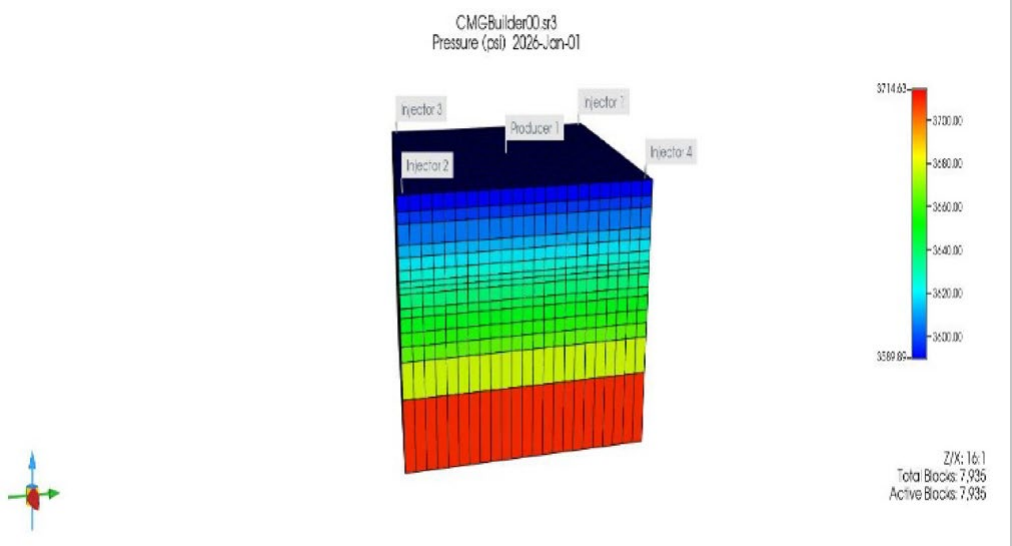
Uniform pressure communication and oil saturation maps achieved in the ¼ five-spot pattern after combined CO_2_-polymer injection.

The ¼ five-spot pattern combined with polymer injection is expected to substantially enhance areal sweep efficiency by reducing the tendency of injected fluids to bypass oil along high-permeability flow paths toward the producer. The improved mobility control provided by the polymer, together with selective perforation, also mitigates gravity override of CO_2_ and stabilizes vertical displacement^106^. These effects are evident in CMG-IMEX results through more uniform oil saturation distributions, a reduction in unswept zones, and improved pressure communication across the perforated layers (layers 9–11), as shown in Fig. 14, indicating a more efficient and balanced reservoir drainage.

The simulation study was developed as an upscaling and validation framework, with model inputs derived directly from experimental coreflood data. Several key parameters were calibrated based on laboratory observations, including: (1) endpoint relative permeabilities and saturation limits obtained from coreflood mass balance analysis; (2) the polymer viscosity–concentration relationship determined from rheological measurements; (3) residual oil saturation after polymer flooding (S_orp_) and after CO_2_ flooding (S_orpc_); and (4) relative permeability curves adjusted to reproduce the experimentally observed pressure drop and recovery behavior. The simulation outcomes exhibited strong qualitative agreement with experimental trends. Both approaches confirmed that a xanthan gum concentration of 1.5 g/L provides optimal performance, that CO_2_ injection followed by polymer flooding yields the highest recovery, and that hybrid injection strategies improve sweep efficiency and delay breakthrough. Quantitatively, the simulation slightly overpredicted oil recovery by approximately 2–3%, which can be attributed to the simplified, homogeneous nature of the numerical model compared to the inherent heterogeneity of the experimental system. Overall, this calibration strategy ensures that the simulation results represent a realistic extension of laboratory findings rather than purely theoretical predictions. The combined injection of CO_2_ and xanthan gum polymer exhibits a strong synergistic effect by simultaneously enhancing macroscopic sweep efficiency and microscopic displacement mechanisms. Polymer injection improves mobility control and delays water breakthrough, while CO_2_ reduces oil viscosity and residual oil saturation, resulting in higher cumulative oil recovery and more sustainable production performance. In addition to technical benefits, this integrated EOR approach offers economic advantages through reduced water handling and treatment requirements, improved reservoir management via pressure support, and demonstrates strong applicability for mature oil fields experiencing high water cut.

## Conclusion

This study systematically investigated the effectiveness of combining xanthan gum polymer flooding with carbon dioxide (CO_2_) injection to enhance oil recovery under reservoir-representative conditions. Based on detailed rheological characterization, viscoelastic analysis, and sand-pack flooding experiments, the following key conclusions can be drawn:

1. Rheological suitability of xanthan gum

Xanthan gum solutions exhibited pronounced shear-thinning behavior and strong viscoelastic characteristics across the investigated concentration range (1.0–2.5 g/L). This non-Newtonian response is highly advantageous for EOR applications, as it ensures good injectivity under high shear conditions near the wellbore while maintaining elevated viscosity and elasticity under low-shear reservoir conditions.

2. Optimal polymer concentration

Among all tested concentrations, **1.5 g/L xanthan gum consistently demonstrated the most favorable performance**. This concentration achieved an optimal balance between viscosity enhancement, elastic strength, and flowability. Lower concentrations provided insufficient mobility control, while higher concentrations resulted in diminishing rheological benefits and potential injectivity limitations due to excessive chain entanglement and molecular crowding.

3. Viscoelastic contribution to mobility control

Frequency sweep measurements confirmed that xanthan gum solutions behave as weak viscoelastic gels with dominant elastic response (G′ > G″). The elastic nature of the polymer plays a critical role in mobility control by generating normal stresses that help mobilize trapped oil and stabilize displacement fronts. The 1.5 g/L solution exhibited the most effective viscoelastic response, supporting both microscopic displacement efficiency and macroscopic sweep efficiency.

4. Flooding performance and recovery mechanisms

Sand-pack flooding experiments demonstrated that water flooding recovered approximately 70% OOIP, leaving a significant fraction of residual oil. CO_2_ flooding alone increased recovery to about 83.3% OOIP but remained limited by early gas breakthrough. Polymer flooding improved sweep efficiency, with a maximum recovery of approximately 81.3% OOIP achieved at 1.5 g/L xanthan gum.

5. Synergistic effect of polymer–CO_2_ flooding

Hybrid injection strategies significantly outperformed single-method EOR approaches. Polymer flooding followed by CO_2_ injection increased recovery to approximately 89.3% OOIP, while the reverse sequence-CO_2_ flooding followed by 1.5 g/L xanthan gum injection, yielded the highest recovery of about 94.3% OOIP. This superior performance arises from the complementary mechanisms of CO_2_ (oil swelling, viscosity reduction, and IFT lowering) and polymer flooding (mobility control, sweep improvement, and flow redistribution).

6. Injection sequence importance

The results clearly demonstrate that injection order is a critical design parameter. Initiating flooding with CO_2_ mobilizes residual oil, while subsequent polymer injection effectively controls gas mobility, blocks high-permeability channels, and redirects flow toward unswept zones. This sequence maximizes both microscopic and macroscopic displacement efficiencies. The combined xanthan gum polymer and CO_2_ flooding is a technically effective and economically promising EOR strategy for mature sandstone reservoirs. The use of an environmentally friendly biopolymer at an optimized concentration (1.5 g/L) provides robust mobility control without the need for costly cosolvents or complex chemical formulations. In addition to significantly enhancing oil recovery, this hybrid approach improves CO_2_ utilization efficiency and supports long-term carbon management objectives. The insights gained from this study provide a strong experimental foundation for the design and optimization of polymer-assisted CO_2_ flooding schemes at the field scale.

7. Numerical simulations using the CMG-IMEX

Numerical simulations conducted using the CMG-IMEX reservoir simulator corroborated the experimental observations and extended the analysis to field-scale conditions. Simulation results confirmed improved mobility control, delayed water and gas breakthrough, enhanced sweep efficiency, and higher cumulative oil recovery for the combined polymer–CO_2_ flooding schemes. The simulations further highlighted the critical importance of injection sequence, showing that initiating flooding with CO_2_ enhances oil mobilization, while subsequent polymer injection effectively controls gas mobility, blocks high-permeability flow paths, and redirects the displacement toward unswept regions.

## Supplementary Information

Below is the link to the electronic supplementary material.

Supplementary Material 1

## Data Availability

The raw data used and analyzed during the current study are available from the corresponding author upon request.
