# Supplementary material for: A numerical and experimental approach to oil recovery performances during combined xanthan gum and carbon dioxide flooding
Source: Sci Rep. 2026 May 1;16:14018. doi: 10.1038/s41598-026-49640-7 (PMC13134968; doi:10.1038/s41598-026-49640-7)
Supplement: Supplementary file 1 — Supplementary Material 1 [file 41598_2026_49640_MOESM1_ESM.docx]

**A Numerical and Experimental Approach to oil recovery performances during combined Xanthan Gum and Carbon dioxide flooding.**

**A.N. El-hoshoudy ^1,2*^ , and E.M. Mansour ^1,2*^**

^1^PVT lab, Production Department, Egyptian Petroleum Research Institute, 11727, Cairo, Egypt.

^2^ PVT service center, Egyptian Petroleum Research Institute, 11727, Cairo, Egypt.

Corresponding author:

A.N. El-hoshoudy; E-mail: [azizchemist@yahoo.com](mailto:azizchemist@yahoo.com); abdelaziz.nasr@epri.sci.eg

E.M. Mansour, E-mail: [emanmansour84@yahoo.com](mailto:emanmansour84@yahoo.com)

Table S1: The composition analysis of the stock tank oil

| **No.** | **Component** | **MW** | **Mole%** |
| --- | --- | --- | --- |
| **1** | **Hydrogen sulphide** | **34.080** | **0.000** |
| **2** | **Carbon dioxide** | **44.010** | **0.000** |
| **3** | **Nitrogen** | **28.013** | **0.000** |
| **4** | **Methane** | **16.043** | **0.000** |
| **5** | **Ethane** | **30.070** | **0.054** |
| **6** | **Propane** | **44.097** | **0.737** |
| **7** | **I-Butane** | **58.123** | **1.105** |
| **8** | **n-Butane** | **58.123** | **1.549** |
| **9** | **neo-Pentane** | **72.150** | **0.000** |
| **10** | **I-Pentane** | **72.150** | **1.936** |
| **11** | **n-Pentane** | **72.150** | **2.357** |
| **12** | **Hexanes** | **86.180** | **9.922** |
| **13** | **Me-Cyclo-Pentane** | **84.160** | **1.683** |
| **14** | **Benzene** | **78.110** | **0.090** |
| **15** | **Cyclo-Hexane** | **84.160** | **3.099** |
| **16** | **Heptanes** | **100.200** | **6.765** |
| **17** | **Me-Cyclo-Hexane** | **98.190** | **2.025** |
| **18** | **Toluene** | **92.140** | **2.813** |
| **19** | **Octanes** | **114.230** | **5.506** |
| **20** | **Ethyle-Benzene** | **106.170** | **1.607** |
| **21** | **Meta/Para-Xylene** | **106.170** | **2.411** |
| **22** | **Ortho-Xylene** | **106.170** | **1.527** |
| **23** | **Nonanes** | **128.260** | **4.018** |
| **24** | **Tri-Me-Benzene** | **120.190** | **0.552** |
| **25** | **Decanes** | **142.285** | **4.510** |
| **26** | **Undecanes** | **147.000** | **4.330** |
| **27** | **Dodecanes** | **161.000** | **4.149** |
| **28** | **Tridecanes** | **175.000** | **3.969** |
| **29** | **Tetradecanes** | **190.000** | **3.698** |
| **30** | **Pentadecanes** | **206.000** | **3.518** |
| **31** | **Hexadecanes** | **222.000** | **3.247** |
| **32** | **Heptadecanes** | **237.000** | **2.977** |
| **33** | **Octadecanes** | **251.000** | **2.616** |
| **34** | **Nondecanes** | **263.000** | **2.435** |
| **35** | **Eicosanes** | **275.000** | **2.165** |
| **36** | **Heneicosanes** | **291.000** | **1.894** |
| **37** | **Docosanes** | **305.000** | **1.714** |
| **38** | **Tricosanes** | **318.000** | **1.624** |
| **39** | **Tetracosanes** | **331.000** | **1.353** |
| **40** | **Pentcosanes** | **345.000** | **1.173** |
| **41** | **Hexacosanes** | **359.000** | **0.911** |
| **42** | **Heptacosanes** | **374.000** | **0.812** |
| **43** | **Octacosanes** | **388.000** | **0.722** |
| **44** | **Nonacosanes** | **402.000** | **0.631** |
| **45** | **Triacosanes** | **416.000** | **0.541** |
| **46** | **Hentriacosanes** | **430.000** | **0.451** |
| **47** | **Dotriacosanes** | **444.000** | **0.361** |
| **48** | **Tritriacosanes** | **458.000** | **0.162** |
| **49** | **Tetratriacosanes** | **472.000** | **0.081** |
| **50** | **Pentatriacosanes** | **486.000** | **0.048** |
| **51** | **Hexatriacosanes Plus** | **506.970** | **0.150** |
|  |  |  |  |
|  | **Total=** | | **100** |
